# A single-cell transcriptomic atlas of primate pancreatic islet aging

**DOI:** 10.1093/nsr/nwaa127

**Published:** 2020-06-10

**Authors:** Jingyi Li, Yuxuan Zheng, Pengze Yan, Moshi Song, Si Wang, Liang Sun, Zunpeng Liu, Shuai Ma, Juan Carlos Izpisua Belmonte, Piu Chan, Qi Zhou, Weiqi Zhang, Guang-Hui Liu, Fuchou Tang, Jing Qu

**Affiliations:** State Key Laboratory of Membrane Biology, Institute of Zoology, Chinese Academy of Sciences, Beijing 100101, China; National Laboratory of Biomacromolecules, CAS Center for Excellence in Biomacromolecules, Institute of Biophysics, Chinese Academy of Sciences, Beijing 100101, China; Beijing Institute for Brain Disorders, Advanced Innovation Center for Human Brain Protection, National Clinical Research Center for Geriatric Disorders, Xuanwu Hospital Capital Medical University, Beijing 100053, China; University of Chinese Academy of Sciences, Beijing 100049, China; Beijing Advanced Innovation Center for Genomics, Biomedical Pioneering Innovation Center, College of Life Sciences, Peking University, Beijing 100871, China; Ministry of Education Key Laboratory of Cell Proliferation and Differentiation, Beijing 100871, China; Peking-Tsinghua Center for Life Sciences, Academy for Advanced Interdisciplinary Studies, Peking University, Beijing 100871, China; State Key Laboratory of Membrane Biology, Institute of Zoology, Chinese Academy of Sciences, Beijing 100101, China; University of Chinese Academy of Sciences, Beijing 100049, China; State Key Laboratory of Membrane Biology, Institute of Zoology, Chinese Academy of Sciences, Beijing 100101, China; University of Chinese Academy of Sciences, Beijing 100049, China; Institute for Stem Cell and Regeneration, Chinese Academy of Sciences, Beijing 100101, China; State Key Laboratory of Membrane Biology, Institute of Zoology, Chinese Academy of Sciences, Beijing 100101, China; Beijing Institute for Brain Disorders, Advanced Innovation Center for Human Brain Protection, National Clinical Research Center for Geriatric Disorders, Xuanwu Hospital Capital Medical University, Beijing 100053, China; University of Chinese Academy of Sciences, Beijing 100049, China; Institute for Stem Cell and Regeneration, Chinese Academy of Sciences, Beijing 100101, China; The MOH Key Laboratory of Geriatrics, Beijing Hospital, National Center of Gerontology, Beijing 100730, China; State Key Laboratory of Stem Cell and Reproductive Biology, Institute of Zoology, Chinese Academy of Sciences, Beijing 100101, China; University of Chinese Academy of Sciences, Beijing 100049, China; State Key Laboratory of Membrane Biology, Institute of Zoology, Chinese Academy of Sciences, Beijing 100101, China; University of Chinese Academy of Sciences, Beijing 100049, China; Institute for Stem Cell and Regeneration, Chinese Academy of Sciences, Beijing 100101, China; Gene Expression Laboratory, Salk Institute for Biological Studies, La Jolla, CA 92037, USA; Beijing Institute for Brain Disorders, Advanced Innovation Center for Human Brain Protection, National Clinical Research Center for Geriatric Disorders, Xuanwu Hospital Capital Medical University, Beijing 100053, China; State Key Laboratory of Stem Cell and Reproductive Biology, Institute of Zoology, Chinese Academy of Sciences, Beijing 100101, China; University of Chinese Academy of Sciences, Beijing 100049, China; Institute for Stem Cell and Regeneration, Chinese Academy of Sciences, Beijing 100101, China; University of Chinese Academy of Sciences, Beijing 100049, China; Institute for Stem Cell and Regeneration, Chinese Academy of Sciences, Beijing 100101, China; CAS Key Laboratory of Genomic and Precision Medicine, Beijing Institute of Genomics, Chinese Academy of Sciences, Beijing 100101, China; China National Center for Bioinformation, Beijing 100101, China; State Key Laboratory of Membrane Biology, Institute of Zoology, Chinese Academy of Sciences, Beijing 100101, China; National Laboratory of Biomacromolecules, CAS Center for Excellence in Biomacromolecules, Institute of Biophysics, Chinese Academy of Sciences, Beijing 100101, China; Beijing Institute for Brain Disorders, Advanced Innovation Center for Human Brain Protection, National Clinical Research Center for Geriatric Disorders, Xuanwu Hospital Capital Medical University, Beijing 100053, China; University of Chinese Academy of Sciences, Beijing 100049, China; Institute for Stem Cell and Regeneration, Chinese Academy of Sciences, Beijing 100101, China; Beijing Advanced Innovation Center for Genomics, Biomedical Pioneering Innovation Center, College of Life Sciences, Peking University, Beijing 100871, China; Ministry of Education Key Laboratory of Cell Proliferation and Differentiation, Beijing 100871, China; Peking-Tsinghua Center for Life Sciences, Academy for Advanced Interdisciplinary Studies, Peking University, Beijing 100871, China; State Key Laboratory of Stem Cell and Reproductive Biology, Institute of Zoology, Chinese Academy of Sciences, Beijing 100101, China; University of Chinese Academy of Sciences, Beijing 100049, China; Institute for Stem Cell and Regeneration, Chinese Academy of Sciences, Beijing 100101, China

**Keywords:** islet, β-cell, aging, single-cell RNA sequencing, primate

## Abstract

Aging-related degeneration of pancreatic islet cells contributes to impaired glucose tolerance and diabetes. Endocrine cells age heterogeneously, complicating the efforts to unravel the molecular drivers underlying endocrine aging. To overcome these obstacles, we undertook single-cell RNA sequencing of pancreatic islet cells obtained from young and aged non-diabetic cynomolgus monkeys. Despite sex differences and increased transcriptional variations, aged β-cells showed increased unfolded protein response (UPR) along with the accumulation of protein aggregates. We observed transcriptomic dysregulation of UPR components linked to canonical ATF6 and IRE1 signaling pathways, comprising adaptive UPR during pancreatic aging. Notably, we found aging-related β-cell-specific upregulation of HSP90B1, an endoplasmic reticulum-located chaperone, impeded high glucose-induced insulin secretion. Our work decodes aging-associated transcriptomic changes that underlie pancreatic islet functional decay at single-cell resolution and indicates that targeting UPR components may prevent loss of proteostasis, suggesting an avenue to delaying β-cell aging and preventing aging-related diabetes.

## Teaser

This single-cell transcriptomic study of aged pancreatic islet cells from cynomolgus monkeys reveals loss of proteostasis as a hallmark of β-cell aging.

## INTRODUCTION

Pancreatic islet cells are vital regulators of glucose metabolism and their decay during aging leads to decreased glucose tolerance and even diabetes [1]. Mounting pieces of evidence suggest that aging can cause increased islet mass, impaired islet turnover and increased transcriptional noise in mammalian pancreatic islets [2]. Senescent cells accumulate in pancreatic islets with age, as well as in type 2 diabetes (T2D) [3]. Conversely, clearance of senescent islet cells improves pancreatic endocrine function and restores glucose homeostasis [4], strongly supporting a link between aging, functional failure of islet cells and diabetes. Therefore, a better understanding of molecular changes in aged islet cells may help preserve or regenerate endocrine function, opening up new therapeutic opportunities to inhibit the progression of diabetes in the context of aging.

Endocrine cells are spherically clustered into the islets of Langerhans, and constitute only about 1%–4% of total pancreas mass [5]. Pancreatic islets consist of four major endocrine cell types: glucagon-producing α-cells, insulin-producing β-cells, somatostatin-producing δ-cells and polypeptide-producing PP-cells [5,6]. These cells interact with each other and regulate glucose homeostasis in a multi-hormonal manner [7]. The cellular composition and topological structure of islets vary across different mammalian species [5]. Human islet biology is still poorly understood due to the limitations in sample availability and ethical concerns. Non-human primates (NHPs) such as cynomolgus monkeys are similar to humans in terms of pancreatic structure and diabetes susceptibility, providing comparative models to study primate islet aging. Indeed, the spontaneous occurrence of aging-related insulin resistance and diabetes, as seen in diabetes patients, is observed in monkeys [8]. Thus, obtaining and analyzing islets isolated from cynomolgus monkeys will enable a better understanding of the mechanism underlying the etiology of aging-related diabetes.

One obstacle that impedes a deeper understanding of islet biology is the high variability between islet cells. Endocrine cells are highly heterogeneous in properties, including hormone secretion and glucose responsiveness [9,10]. Heterogeneity is also observed in the expression of aging markers between and within islets in the same pancreas upon aging. Thus, conventional analysis of a whole islet or sorted cell populations may mask subtle changes within certain cell populations that drive functional heterogeneity [11]. Recent advances in single-cell RNA sequencing (scRNA-seq) have allowed the collection of transcriptomic data from individual endocrine cells and profiling of cell-type-specific changes during the emergence of metabolic diseases. Using the approach, scRNA-seq studies of human pancreas have been reported, supporting increased transcriptional noise and loss of cell identity during aging [11–13]. However, the critical molecular drivers underlying islet cell functional decline during aging amid transcriptional heterogeneity remain unclear. Identifying the cell type particularly vulnerable to aging and uncovering the molecular changes occurring during pancreatic islet cell aging are critical to the development of accurate interventions against aging-related diseases [14,15].

To address this gap, we assembled a pancreatic islet aging atlas for non-diabetic cynomolgus monkeys at single-cell resolution, and identified endocrine α-, β-, δ- and PP-cells. Cell-type-specific effects of aging on gene expression signatures were analyzed, supporting increased cell-to-cell transcriptional noise in α-cells and β-cells during aging. Despite the existence of gender dimorphism and transcriptional noise, the unfolded protein response (UPR) emerged as a major pathway affected by aging specifically in β-cells. The analysis revealed escalated expression of UPR genes in canonical activating transcription factor 6 (ATF6) and inositol-requiring enzyme 1 (IRE1) signaling pathways, consistent with increased aggresomal signals in pancreatic islets of old individuals. *HSP90B1*, an endoplasmic reticulum (ER) chaperone, was one of the most upregulated genes and was increased specifically in aged β-cells. Upon glucose exposure, exogenous expression of *HSP90B1* in pancreatic islet cells resulted in compromised insulin secretion, suggesting that UPR proteins play a crucial role in the regulation of insulin secretion and glucose sensing. Our study provides a foundational resource of an NHP pancreatic islet aging atlas, identifies loss of proteostasis as a primary hallmark of β-cell aging, and therefore provides new intervention targets for aging-related pancreatic diseases.

## RESULTS

### Single-cell RNA sequencing of pancreatic islets from non-human primates

We selected eight young (4–6 years old) and eight old (18–21 years old) healthy cynomolgus monkeys, analogous to approximately 20- and 70-year-old humans, respectively (Supplementary Fig. S1A). To evaluate pancreatic function in hormone secretion and glucose control, we measured fasting blood glucose, insulin, c-peptide and glucagon levels in different animal groups, which showed no obvious differences between young and old individuals (Supplementary Fig. S1B and C). Glucose tolerance was weakened in old monkeys, but not statistically significant (Supplementary Fig. S1B). Additionally, an examination of classic features of islet senescence of rodents, including increased islet volume and enlarged islet size, revealed no statistical difference between young and old monkeys (Supplementary Fig. S1D) [1,10,16]. Altogether, we did not detect apparent differences in pancreatic islet structure or secretory function between young and old monkeys, allowing us to monitor the molecular effect of aging in advance of the appearance of diabetic phenotypes.

**Figure 1.**
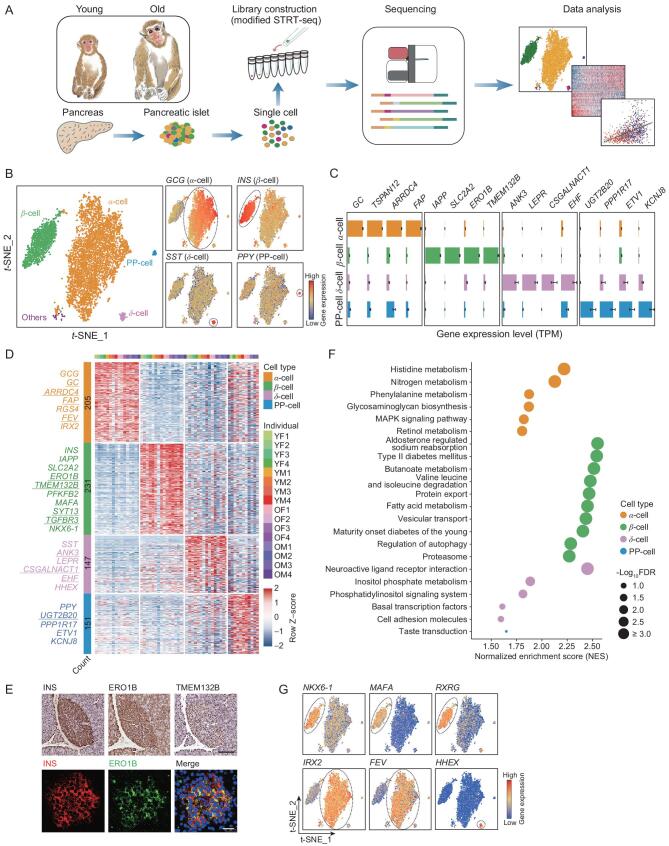
Single-cell transcriptomic atlas of pancreatic islet aging in cynomolgus monkeys. (A) Workflow showing the procedure of scRNA-seq of monkey pancreatic islets. (B) *t*-SNE plot showing pancreatic islet cell types (left) and expression levels of classical marker genes for each cell type (right). The corresponding cell type is denoted in circles. (C) Bar charts showing the expression levels of representative genes in different cell types. All expression levels are measured with the same scale. Data are shown as mean ± SEM. (D) Heatmap showing the row scaled expression levels (Continued). of cell-type-specific marker genes for each cell type. Representative genes are shown on the left and novel marker genes are underlined. Color bars at the top of the heatmap indicate monkey individuals. The numbers of cell-type-specific marker genes are shown in the left bar. (E) Top, immunohistochemistry staining of INS, ERO1B and TMEM132B in consecutive pancreatic biopsies. Bottom, immunofluorescent staining of INS and ERO1B in pancreatic biopsies. Scale bar, 100 μm. (F) Dot plot showing top-ranked KEGG pathways enriched in different cell types. Pathways are ranked based on the normalized enrichment score (NES) within each cell type, and the nominal *P* value < 0.05 and false discovery rate (FDR) (*q* value) < 0.25. Colors indicate different cell types and size indicates −log_10_(FDR). (G) *t*-SNE plots showing expression levels of key transcriptional factors for each cell type. The corresponding cell type is denoted in circles.

To analyze the cell populations and molecular characteristics of aged primate pancreatic islets, we performed scRNA-seq of islets from cynomolgus monkeys using a modified single-cell tagged reverse transcription (STRT) protocol (Fig. 1A) [17]. After critical cell quality control and filtering, a total of 5 575 single cells were retained in the downstream analyses, and expression of 4 389 genes on average was detected in each cell (Supplementary Figs S1A and S2A; Supplementary Table 1). Unsupervised clustering analysis separated different cell types into distinct clusters with the absence of individual heterogeneity (Fig. 1B; Supplementary Fig. S2B; Supplementary Table 1). All major cell types were identified, based on specific cell marker expression, including *GCG* (α-cell), *INS* (β-cell), *SST* (δ-cell) and *PPY* (PP-cell) (Fig. 1B). The number of cell-type-specific genes found in β-cells surpasses that of other cell types, indicative of their highly specialized function (Fig. 1C and D; Supplementary Fig. S2C; Supplementary Table 1). A plethora of known β-cell markers were found, including *IAPP* encoding Islet Amyloid Polypeptide,which is another hormone secreted from β-cells, and *SLC2A2* (also known as *GLUT2*) responsible for glucose uptake in β-cells (Fig. 1C and D; Supplementary Fig. S2C and D) [18]. In addition, *ERO1B*, a gene that encodes endoplasmic reticulum oxidoreductase 1 beta [19], and *TMEM132B* [20], which functions in cellular adhesion, were identified and experimentally verified as novel markers for β-cells (Fig. 1C–E). We also identified several novel cell-type-specific genes for α-cells (e.g. *TSPAN12* and *ARRDC4*), δ-cells (e.g. *ANK3*, *EHF* and *CSGALNACT1*) and PP-cells (e.g. *UGT2B20*) (Fig. 1C and D; Supplementary Fig. S2D). Gene Ontology (GO) analysis indicated that these cell-type-specific markers were related to the unique metabolic function of these cells (Supplementary Fig. S2C). Furthermore, gene set enrichment analysis (GSEA) based on Kyoto Encyclopedia of Genes and Genomes (KEGG) pathways showed that compared to non-β-cells, β-cells preferentially expressed genes involved in terms related to T2D, the onset of type 1 diabetes and aldosterone regulated sodium reabsorption, all of which matched the function of β-cells in pancreatic islets (Fig. 1F; Supplementary Table 1). In addition, *PROCR*^+^ cells were recently characterized to be pancreatic islet progenitors in adult mice [21]. We found that a small proportion of endocrine cells expressed this marker in the monkey pancreases (Supplementary Fig. S2E and F).

Among the cell-type-specific markers, we identified several key transcriptional factors that might play important roles in cell-type-specific gene regulatory processes, including *IRX2* and *FEV* in α-cells; *NKX6–1*, *MAFA* and *RXRG* in β-cells; and *HHEX* in δ-cells (Fig. 1G). Many of these transcriptional regulators play a critical role in cell fate determination during endocrine development or trans-differentiation between different islet cell identities [22,23]. Furthermore, dysregulation of these marker genes and transcriptional factors is linked to the onset of diabetes, with many of these genes already recognized as diabetes-causing genes (Supplementary Fig. S2G and H). Altogether, we uncovered cell-type-specific transcriptional signatures with novel marker genes that reflected the functional characteristics of each cell type in pancreatic islets.

### Aging markers and transcriptional noise inducers identified by scRNA-seq of aged pancreatic islets

**Figure 2.**
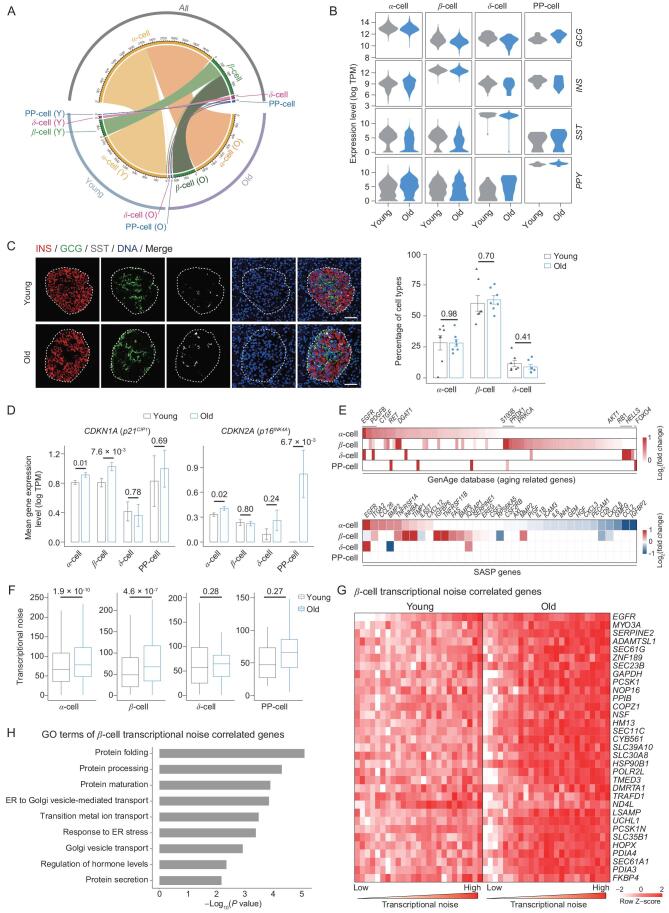
Cell identity changes in pancreatic islet cells during aging. (A) Circos plot showing the corresponding relationship of cell types analyzed based on different datasets. Top semicircle, cell types are analyzed based on all cells. Bottom semicircle, cell types are analyzed based on cells only from young individuals (left) or cells only from old individuals (right). (B) Violin plots showing expression levels of classical marker genes for each cell type in young and old individuals. (C) Immunofluorescent staining of known markers INS, GCG, SST for β-cells, α-cells and δ-cells, respectively (left). Pancreatic islets are circled with dashed lines. Bar chart showing the cell percentages of different cell types in young and old monkeys (right). Scale bar, 50 μm. *n* = 7 monkeys per group. Data are shown as mean ± SEM. *P* values are indicated (two-tailed *t*-test). (D) Bar charts showing the mean gene expression level of two classic aging markers (*CDKN1A* and *CDKN2A*) in each cell type. *P* values are indicated (two-tailed *t*-test). Data are shown as mean ± SEM. (E) Heatmaps showing the fold change of upregulated aging-related genes in the GenAge dataset (top) and of SASP genes (bottom). Only the genes differentially regulated in at least one type of cells between young and old individuals are analyzed. (F) Boxplots showing the transcriptional noise analysis in each cell type. *P* values are indicated (two-tailed *t*-test). (G) Heatmap showing the row scaled expression level of genes with high Pearson's correlation coefficients (correlation coefficient > 0.6 and FDR < 0.05) between transcriptional noise and expression levels in β-cells. The heatmap is separated into two groups, cells collected from young individuals and cells from old individuals, and bins are arranged based on the transcriptional noise rank in each group. (H) Bar chart showing representative GO terms of genes whose expression correlated with transcriptional noise in β-cells. Cell identity changes in pancreatic islet cells during aging. (A) Circos plot showing the corresponding relationship of cell types analyzed based on different datasets. Top semicircle, cell types are analyzed based on all cells. Bottom semicircle, cell types are analyzed based on cells only from young individuals (left) or cells only from old individuals (right). (B) Violin plots showing expression levels of classical marker genes for each cell type in young and old individuals. (C) Immunofluorescent staining of known markers INS, GCG, SST for β-cells, α-cells and δ-cells, respectively (left). Pancreatic islets are circled with dashed lines. Bar chart showing the cell percentages of different cell types in young and old monkeys (right). Scale bar, 50 μm. *n* = 7 monkeys per group. Data are shown as mean ± SEM. *P* values are indicated (two-tailed *t*-test). (D) Bar charts showing the mean gene expression level of two classic aging markers (*CDKN1A* and *CDKN2A*) in each cell type. *P* values are indicated (two-tailed *t*-test). Data are shown as mean ± SEM. (E) Heatmaps showing the fold change of upregulated aging-related genes in the GenAge dataset (top) and of SASP genes (bottom). Only the genes differentially regulated in at least one type of cells between young and old individuals are analyzed. (F) Boxplots showing the transcriptional noise analysis in each cell type. *P* values are indicated (two-tailed *t*-test). (G) Heatmap showing the row scaled expression level of genes with high Pearson's correlation coefficients (correlation coefficient > 0.6 and FDR < 0.05) between transcriptional noise and expression levels in β-cells. The heatmap is separated into two groups, cells collected from young individuals and cells from old individuals, and bins are arranged based on the transcriptional noise rank in each group. (H) Bar chart showing representative GO terms of genes whose expression correlated with transcriptional noise in β-cells.

Next, we asked whether aging altered cell identity or cell-type distribution in pancreatic islets. The hormonal expression pattern and transcriptional signatures of marker genes in each cell type were undisturbed in the aged islets, indicating that cell identity itself was not changed during aging (Fig. 2A and B). Moreover, sequencing data and immunofluorescence staining indicated no differences in the proportions of islet cell types presented during aging (Fig. 2C; Supplementary Fig. S3A–D).

To evaluate the senescent state of islets from old monkeys, we examined the expression of the senescence markers *CDKN1A* (*p21^CIP1^*) and *CDKN2A* (*p16^INK4A^*) in different islet cell types [10,24,25]. Consistent with the previous study [13], increased *CDKN1A* and *CDKN2A* expression were revealed in aged α-cells or β-cells (Fig. 2D). Furthermore, more aging-related genes collected in the GenAge dataset and genes involved in senescence-associated secretory phenotype (SASP) were also dysregulated in aged endocrine cells, especially in α-cells and β-cells (Fig. 2E) [26]. These results implied that islets in aged monkeys were enriched with senescent endocrine cells and that α-cells and β-cells appeared more vulnerable to aging compared to other pancreatic islet cell types.

To further analyze aging-related perturbations in the transcriptome, we first measured transcriptional noise in different cell types (see Supplementary Information for details) [13]. Results indicated increased transcriptional noise in aged α-cells (two-tailed Student's *t*-test *P* = 1.9 × 10^−10^) and β-cells (*P* = 4.6 × 10^−7^), but not δ-cells (*P* = 0.3) or PP-cells (*P* = 0.3), compared to young counterparts, suggesting a cell-type-specific age-dependent increase in transcriptional noise (Fig. 2F). To identify genes whose transcriptional fluctuations were accompanied with increased transcriptional noise, we calculated the Pearson's correlation (Pearson's correlation coefficient > 0.6 and false discovery rate [FDR] < 0.05) between gene expression levels and transcriptional noise in α-cells and β-cells (Fig. 2G; Supplementary Fig. S3E). GO enrichment analysis further showed that genes involved in protein folding (including *HSP90B1*, *PDIA3* and *PDIA4*), protein processing and maturation were the dominant genes underlying age-upregulated transcriptional noise in β-cells (Fig. 2H). These analyses highlighted the importance of protein processing in controlling cellular heterogeneity during islet aging.

### Systemic portrayal of the transcriptomic landscape of aged α-cells and β-cells

**Figure 3.**
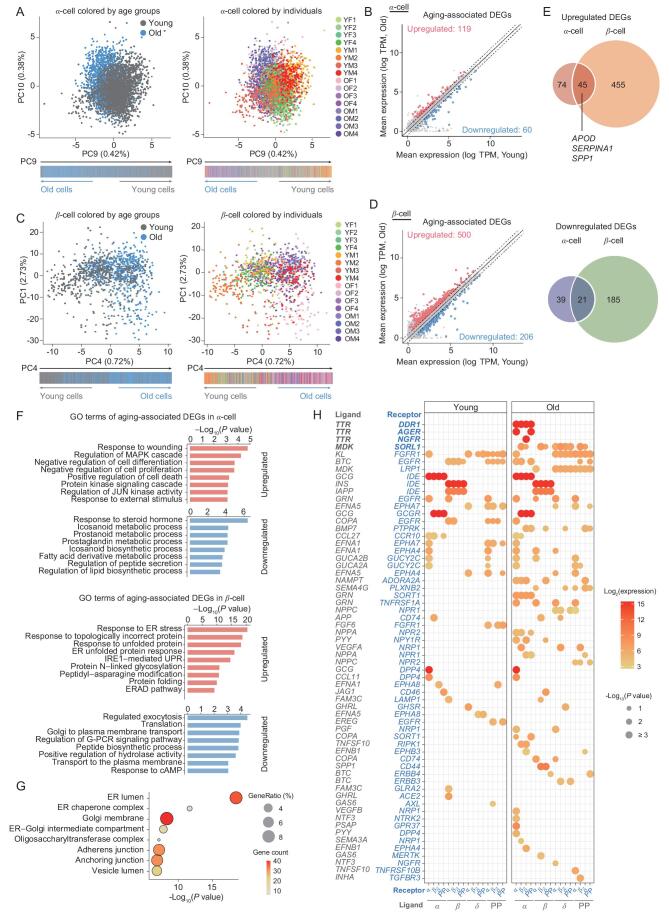
Transcriptomic characteristics in aged α-cells and β-cells. (A) PCA plots showing the distribution of α-cells colored by age groups (left) and individuals (right). Bars showing the distribution of α-cells along PC9 dimension at the bottom. (B) Scatter plot for upregulated and downregulated aging-associated DEGs in aged α-cells. Red and blue points indicate upregulated and downregulated aging-associated DEGs, respectively; gray points indicate other genes. The numbers of DEGs are shown. (C) PCA plots showing the distribution of β-cells colored by age groups (left) and individuals (right). Bars showing the distribution of β-cells along PC4 dimension at the bottom. (D) Scatter plot for upregulated and downregulated aging-associated DEGs in aged β-cells. Red and blue points indicate upregulated and downregulated aging-associated DEGs, respectively; gray points indicate other genes. The numbers of DEGs are shown. (E) Venn diagrams showing the comparison of aging-associated DEGs in α-cells and β-cells. Upper and lower panels correspond to upregulated and downregulated aging-associated DEGs, respectively. Representative shared upregulated DEGs are indicated. (F) Bar charts showing representative GO terms of upregulated or downregulated aging-associated DEGs in aged α- (top) and β-cells (bottom). (G) Dot plot showing top-ranked GO (Cellular Components) terms of upregulated aging-associated DEGs in β-cells. Dot color indicates the number of genes and size indicates the GeneRatio corresponding to terms. (H) Dot plots showing the ligand-receptor interaction pattern in different cell-cell pairs. Dot color indicates the mean expression level in ligand-receptor pairs and dot size indicates the statistical significance. Gray words indicate the ligands (row) expressed in the corresponding cell type (column), and blue words indicate the receptors (row) expressed in the corresponding cell type (column).

Could we uncover meaningful transcriptomic changes underlying aged endocrine cells given the increased transcriptional noise and individual variation during aging? To answer this question, we sought to systemically analyze aging-associated differentially expressed genes (DEGs) between young and old α-cells and β-cells by performing the principal component analysis (PCA) to separate young and old cells into distinct principal component (PC) dimensions (see Supplementary Information for details) [27]. For α-cells, along the PC1 to PC8 axis, transcriptional intervention from individual variations was gradually excluded, allowing us to divide α-cells into two prominent groups of cells corresponding to young and old individuals (Supplementary Fig. S4A). Young α-cells were inclined to have high PC9 scores whereas old α-cells had low PC9 scores, which were highly consistent across all the individuals and were therefore not attributable to technical or batch effects (Fig. 3A; Supplementary Fig. S4A; Supplementary Table 2). We defined young and old α-cells by combining information from the principal component analysis and the aging group information of every single cell. Finally, we obtained 1443 young α-cells and 1397 old α-cells. Old α-cells with low PC9 scores showed high expression of 119 genes, and low expression of 60 genes compared to young α-cells with high PC9 scores. We referred to these genes as upregulated aging-associated DEGs and downregulated aging-associated DEGs in α-cells, respectively (Fig. 3B; Supplementary Table 2). The same PCA strategy was applied to β-cells, enabling separation of young (369 cells) and old (446 cells) β-cells along the PC4 axis (Fig. 3C; Supplementary Fig. S4A; Supplementary Table 2). A fraction of aged β-cells with high PC4 scores displayed 500 upregulated aging-associated DEGs and 206 downregulated aging-associated DEGs (Fig. 3D; Supplementary Table 2). Aged δ-cells and PP-cells were not discriminated by this method, probably due to the limited number of these two types of cells. Finally, we sought to identify aging-associated DEGs by directly comparing all cells collected from young and old monkeys (Supplementary Table 3; see Supplementary Information for details). The results showed that the majority of aging-associated DEGs identified by the additional analysis overlapped with DEGs obtained through the PCA strategy (Supplementary Fig. S4B–E).

When we compared aging-associated DEGs between α-cells and β-cells, four times more DEGs were identified in β-cells (706 DEGs) than in α-cells (179 DEGs), and only a small fraction of them overlapped (45 and 21 common upregulated and downregulated DEGs, respectively) (Fig. 3E). We also noticed that genes that encode secretory proteins or cytokines such as *SPP1*, *APOD* and *SERPINA1* were among the upregulated aging-associated DEGs in both aged α-cells and β-cells, suggesting that paracrine interactions may be disrupted during islet aging. Of note, these genes encoding secretion proteins (e.g. *APOD* and *SERPINA1*) were also reported to be increased in aged human serum (Supplementary Fig. S5A) [28], and may therefore be useful as clinical aging markers to help diagnose the extent of islet aging. We then analyzed transcriptional drift with age for each cell type. Upregulated aging-associated DEGs in α-cells were enriched in response to wounding, negative regulation of cell proliferation and positive regulation of cell death (Fig. 3F; Supplementary Table 2). Downregulated aging-associated DEGs of α-cells were enriched in various metabolic processes linked to fatty acid and prostaglandin metabolism, and regulation of peptide secretion (Fig. 3F; Supplementary Table 2), implying that the basic metabolic function of α-cells was compromised during aging.

In aged β-cells, we found decreased expression of genes linked to secretory granule biogenesis [29], such as regulated exocytosis, Golgi to plasma membrane transport and peptide biosynthetic process (Fig. 3F; Supplementary Table 2). Strikingly, upregulated aging-associated DEGs in β-cells were enriched in ER stress and UPR related pathways, with repeated appearance of GO terms like ‘response to ER stress’, ‘protein folding’ and ‘ER to Golgi vesicle-mediated transport’ (Fig. 3F; Supplementary Table 2). In addition, analyses by GO (Cellular Component) also showed that upregulated aging-associated DEGs in β-cells encode proteins highly enriched in ER lumen, ER chaperone complex and Golgi membrane (Fig. 3G). These results underscored a loss of proteostasis along with increased ER stress that activated UPR signaling, which comprised a major molecular change in pancreatic β-cell aging.

To further reveal the dynamic reciprocal interactions between islet cell types and their effects on β-cell function during aging, we built a predicted cellular network based on the expression pattern of potential ligand-receptor pairs in different cell types (Supplementary Fig. S5B) [30,31]. The computerized network revealed that cell-cell communications were more enhanced in old islets than in young islets, especially the interaction between α-cell-expressed ligand TTR and its receptor DDR1 (Fig. 3H). TTR, an etiologic agent associated with aggregation of misfolded proteins and amyloidoses, was highly expressed in aged α-cells, consistent with that in islet cells of type 2 diabetic individuals [32]. In addition, *SORL1*, a gene associated with amyloidogenic processing of amyloid-beta precursor protein (APP) and Alzheimer's disease risk, was upregulated in aged β-cells [33], along with its ligand highly expressed in all four kinds of aged islet cells (Fig. 3H). These results of cell-cell interactions in aged islet suggested that the microenvironments in aged pancreatic islets may contribute to the loss of proteostasis and activation of UPR in aged β-cells. Altogether, comprehensive identification of cell-type-specific transcriptional signature changes in α-cells and β-cells highlighted the loss of proteostasis as an important molecular event during β-cell aging.

### Deciphering changes to the ER stress network in aged β-cells

**Figure 4.**
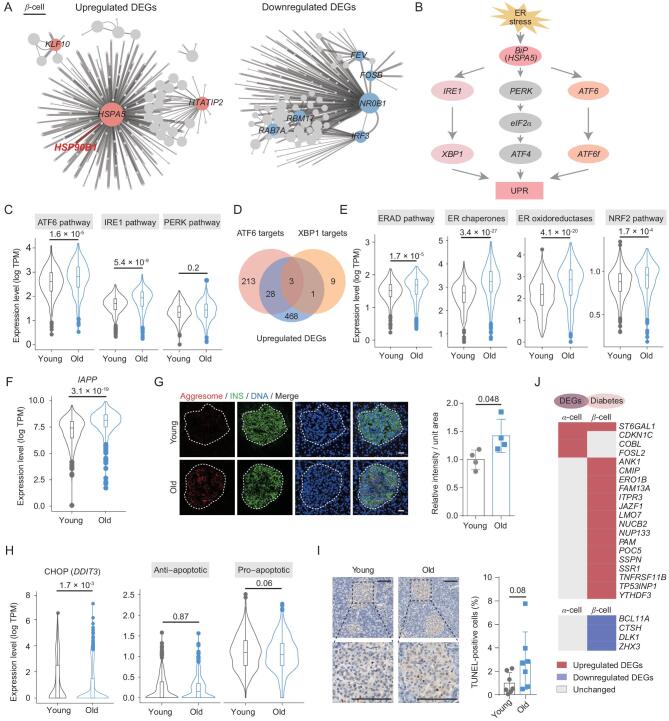
Upregulation of UPR genes in aged β-cells. (A) Regulatory networks visualizing potential key transcriptional regulators in upregulated and downregulated aging-associated DEGs in aged β-cells. Only connections with high weight are retained and node size indicates the number of connections, and nodes with top-ranked size are highlighted in red (upregulated, left) or blue (downregulated, right). (B) A schematic chart showing three main branches and corresponding principal components in the UPR pathway. (C) Violin plots showing the expression level of genes corresponding to UPR pathways in β-cells. *P* values are indicated (two-tailed *t*-test). (D) Venn plot showing the comparison of upregulated aging-associated DEGs in β-cells, ATF6 and XBP1 target genes. (E) Violin plots showing the expression level of genes involved in ERAD pathway, ER chaperones, ER oxidoreductases and NRF2 pathway in β-cells. *P* values are indicated (two-tailed *t*-test). (F) Violin plot showing the expression level of *IAPP* in β-cells. *P* value is indicated (two-tailed *t*-test). (G) Aggresome staining of pancreatic islets in young and old female monkeys (left). Pancreatic islets are circled with dashed lines. Bar chart showing the average aggresome signal levels of pancreatic islets in young and old female monkeys (right). Scale bar, 100 μm. *n* = 4 monkeys for each group. *P* value is indicated (two-tailed *t*-test). Data are shown as mean ± SEM. (H) Violin plots showing the expression level of *DDIT3* (encoding CHOP), anti-apoptotic and pro-apoptotic genes in β-cells. *P* values are indicated (two-tailed *t*-test). (I) TUNEL-staining of young and old pancreases, and (Continued). pancreatic islets are circled with dashed lines. Bar chart showing the percentages of apoptotic cells (TUNEL-positive) in pancreatic islets of young and old monkeys. Scale bar, 100 μm. *n* = 7 monkeys for each group. *P* value is indicated (two-tailed *t*-test). Data are shown as mean ± SEM. (J) Heatmaps showing the distribution of upregulated or downregulated aging-associated DEGs in α-cells and β-cells that overlap with the diabetes gene set.

To identify critical regulators linked to α-cell and β-cell aging, we constructed gene regulatory networks based on aging-associated DEGs (Fig. 4A; Supplementary Fig. S5C). The regulatory network identified *HSPA5* (*BiP*) as a major regulator of upregulated aging-associated DEGs in β-cells (Fig. 4A). This finding was in agreement with the prominent upregulation of UPR pathways found in our GO analysis, as *HSPA5* is a well-known central regulator in response to ER stress. In particular, ER stress was sensed by three ER transmembrane signaling proteins, ATF6, protein kinase RNA (PKR)-like ER kinase (PERK), and IRE1 (Fig. 4B) [34]. We found that components of the ATF6 (two-tailed Student's *t-*test *P* = 1.6 × 10^−5^) and IRE1 (*P* = 5.4 × 10^−9^) pathways were transcriptionally upregulated in aged β-cells, compared to young β-cells (Fig. 4C). These UPR genes included *HSP90B1* and *CALR* activated by ATF6 pathway, and *PDIA6*, *PDIA5*, *DNAJB11*, *DNAJB9* and *DNAJC3* activated by IRE1-XBP1 pathway that encode ER chaperones and Ca^2+^-binding (storage) protein (Supplementary Fig. S5D) [35]. In the list of ATF6 target genes, ∼13% (31 of 244) were identified in our scRNA-seq data as upregulated aging-associated DEGs [36], indicating that ATF6 pathway may have a critical influence on UPR signaling during β-cell aging (Fig. 4D). Converging signals from ATF6 and IRE1 triggered transcriptional upregulation of genes encoding ER-associated degradation (ERAD) components, ER chaperones, oxidoreductases, as well as nuclear factor erythroid 2-related factor 2 (NRF2) pathways members that enhanced cellular capacity to adapt to ER stress (Fig. 4E).

Consistent with the transcriptomic changes, aggresome accumulated in aged β-cells, along with increased expression of islet amyloid polypeptide (*IAPP*) (log_2_(fold change) = 0.67, two-tailed Student's *t*-test *P* = 3.1 × 10^−19^) that spontaneously forms amyloid sheets that would be predicted to disrupt ER membranes (Fig. 4F and G) [37]. To analyze the molecular outcomes of accumulated ER stress and activated UPR, we calculated expression levels of the pro-apoptotic transcription factor CHOP encoded by *DDIT3*, and the Bcl-2 family (Fig. 4H). These pathways herald a UPR switch from the adaptive stage, which eliminates affordable ER stress, to the ‘self-destruct’ stage [38]. This later stage arises in response to chronic or overwhelming stress, and was not activated in aged β-cells (Fig. 4H). Consistently, TUNEL staining showed that cell apoptosis tended to be increased in pancreatic islets, albeit not statistically significant (two-tailed Student's *t*-test *P* = 0.08) (Fig. 4I). Besides, transcriptional changes in genes associated with onset of islet aging related diseases, e.g. diabetes, were present in these aged β-cells (Fig. 4J). Therefore, we may have captured a moment where early adaptive events are present in β-cells, making our data valuable for understanding the etiopathogenesis of diabetes, and for developing measures to prevent the disease.

### Upregulation of *HSP90B1* compromises insulin secretion under glucose stimulation

**Figure 5.**
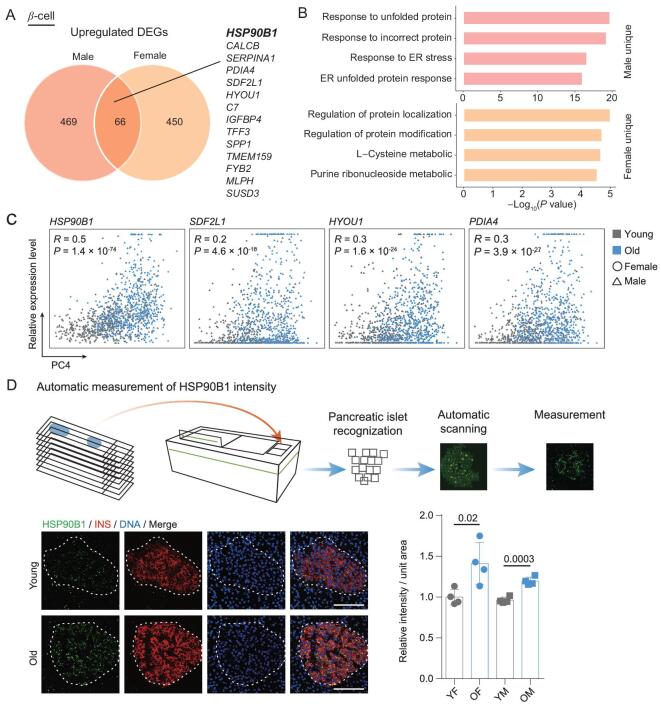
Common upregulation of the UPR gene *HSP90B1* in aged β-cells of both genders. (A) Venn diagram showing the comparison of upregulated aging-associated DEGs in β-cells obtained from male and female monkeys. Representative shared genes are indicated. (B) Bar charts showing representative GO terms of 469 upregulated aging-associated DEGs specific in male-derived aged β-cells (top) and 450 upregulated aging-associated DEGs specific to female-derived (bottom) aged β-cells. (C) Scatter plots showing the relative expression level of four UPR aging-associated DEGs in β-cells along PC4 dimension. Each point indicates a single cell. These genes are common upregulated aging-associated DEGs in both genders. Pearson's correlation coefficient (*R*) and statistical significance (*P*) are indicated. (D) Workflow of section imaging and measurement via Opera Phenix High Content Screening System (upper). Immunofluorescent staining of HSP90B1 and INS (lower left). Pancreatic islets are circled with dashed lines. Bar chart showing the average expression levels of HSP90B1 in pancreatic islets in young female (YF), old female (OF), young male (YM) and old male (OM) monkeys (lower right). Scale bar, 100 μm. *n* = 4 monkeys for each group. *P* values are indicated (two-tailed *t-*test).

To check whether UPR is affected in male and female aged β-cells, we examined single-cell transcriptomic data comparing β-cells from individuals (Fig. 5A and B; Supplementary Table 4). Both female and male β-cells displayed upregulated ER chaperones during aging, including *HSP90B1*, *HYOU1* and *PDIA4* (Fig. 5C). Male β-cells showed more upregulated aging-associated DEGs in the UPR pathway, while female β-cells showed additional upregulation in purine ribonucleotide and L-cysteine metabolic pathways (Fig. 5B). Therefore, UPR was affected in aged β-cells of both genders, although female and male β-cells may regulate different sets of UPR genes.

**Figure 6.**
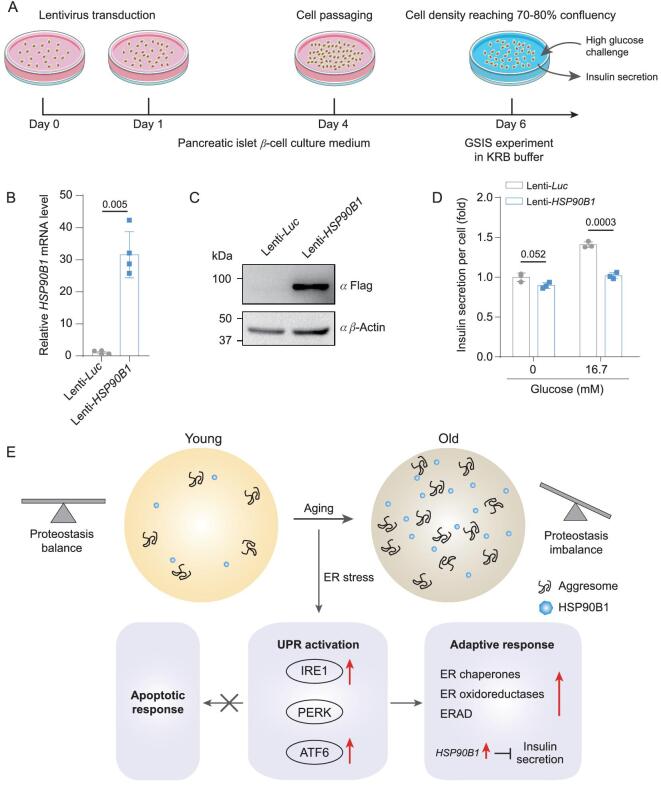
Overexpression of *HSP90B1* impaired insulin secretion in β-cell line under high glucose treatment. (A) Schematic chart showing the workflow of the gene manipulation and GSIS assays. (B) Verification of *HSP90B1* overexpression efficiency in pancreatic islet β-cell line by RT-qPCR. *n* = 4 experimental repeats. *P* value is indicated (two-tailed *t-*test). Data are shown as mean ± SEM. (C) Verification of *HSP90B1* overexpression efficiency in pancreatic islet β-cell line by Western blot. β-Actin is used as a loading control. (D) GSIS assay of pancreatic islet β-cell line expressing *Luc* or *HSP90B1*. *n* = 3 experimental repeats. *P* values are indicated (two-tailed *t-*test). Data are shown as mean ± SEM. (E) Schematic chart showing the accumulation of aggresome and the activation of UPR during β-cell aging.

*HSP90B1*, also known as *GRP94*, was one of the top upregulated aging-associated DEGs in both male and female β-cells (Fig. 5A and C). Immunostaining analyses confirmed that HSP90B1 protein levels were increased in aged β-cells in both genders (Fig. 5D). Although *HSP90B1* is known as a target gene activated by ATF6 pathway during ER stress response, to our knowledge, it has not previously been linked to pancreatic β-cell aging [39]. We overexpressed *HSP90B1* in an insulin-secreting pancreatic β-cell line to estimate the causal effect by increased expression of *HSP90B1* in β-cells (Fig. 6A). The glucose-stimulated insulin secretion (GSIS) assay showed that *HSP90B1* upregulation led to decreased insulin secretion after high glucose stimulation (Fig. 6B–D). Taken together, *HSP90B1* was a candidate target that could lead to interventions that normalize insulin secretion.

## DISCUSSION

Aging is causally linked to a decline in glucose tolerance, even in healthy old subjects [40], but the mechanism remains largely unclear. In this study, we provide a comprehensive single-cell transcriptomic map for non-human primate pancreatic islet cells. First, our database of changes in non-human primate pancreatic cells uncovers altered molecular pathways that are specific to different islet cell types during aging. Second, the loss of proteostasis is identified as a prominent hallmark of β-cell aging. Third, the ATF6 and IRE1 branches of the UPR pathway are upregulated in aged β-cells. Fourth, our data record a molecular status that adaptive UPR is activated instead of pro-apoptotic UPR signaling specifically in aged β-cells. Fifth, changes in the ER stress-related pathways during β-cell aging are conserved between male and female β-cells during aging. Sixth, we reveal that *HSP90B1* is a potential aging effector for β-cells in pancreatic islets of both genders, and that upregulation in cells impairs glucose-induced insulin secretion (Fig. 6E). Collectively, this study provides a valuable resource for discovering diagnostic biomarkers and therapeutic targets for aging-related glucose intolerance.

Single-cell RNA-seq analysis allows the study of cell heterogeneity, and identification of cell states and cell-type-specific gene changes during aging or disease emergence [41]. In the past few years, several studies have used scRNA-seq to analyze human pancreatic islets, enabling a better understanding of islet biology [11–13,42–44]. However, only a few studies have focused on pancreatic islet aging. For instance, scRNA-seq analysis of pancreatic cells from eight human donors spanning about six decades suggests transcriptional instability during islet aging, yet with the detection of only modest age-dependent transcriptional changes that may be related to gender-related heterogeneity and sampling conditions surrounding postmortem human pancreatic islet isolation [13]. By using monkeys that are close to humans as animal models, we were able to control such experiment parameters in a highly stringent and reliable way. Especially when examining heterogeneous islet cells, we used stringent experimental controls that were likely essential to identifying meaningful changes in gene expression signature changes amid increased aging-related transcriptional noise. To our knowledge, this is the first scRNA-seq study that uncovers a link between UPR pathways and β-cell aging through in-depth sequencing by using the modified STRT-seq technology and two mutually reproducible bioinformatics tools.

Recent evidence suggests that toxic IAPP aggregates and genetic mutations caused by the burden of excessive protein aggregation may be linked to β-cell dysfunction and diabetes [45]. Utilizing a transgenic mouse model for monitoring ER stress in pancreas cells, ER stress is increased in old mice compared to young mice [46]. Consistently, our study also found increased *IAPP* expression and protein aggregates in aged β-cells. Loss of proteostasis further aggravates ER stress and triggers UPR signaling [45]. Depending on the intensity and duration of ER stress, UPR signaling is under dynamic control that elicits adaptive or cell death program [38]. How UPR is fine-tuned during aging, and whether and to what extent UPR activation contributes to deteriorating β-cell function and glucose intolerance, await further interrogation of β-cell behavior in the context of the aged pancreas. Our scRNA-seq profiling of aging primate islet cells reveals a comprehensive portrait of UPR pathways that interweave with one another. In the aged β-cells captured in our study, pathways driven by ATF6 and IRE1 signaling were activated, but the PERK branch of UPR signaling, as well as terminal UPR and apoptotic steps, were not mobilized. Of note, PERK and its downstream cell death pathway are activated during type 2 diabetes in response to unresolvable ER stress [47]. Therefore, the upregulation of specific UPR genes may serve as a hallmark of aged β-cells, similar to those in diseased conditions. The maintenance of protein homeostasis is likely to be a critical step for preventing β-cell dysfunction during aging.

Finally, one therapeutic avenue suggested by this study is targeting UPR to prevent glucose intolerance in the pre-diabetic stage. Based on the dissection of UPR signaling at single-cell level, we identified *HSP90B1* as one of the most upregulated genes in aged β-cells of both sexes and of the top transcriptionally heterogeneous genes. *HSP90B1* regulates islet development [48], but its role in β-cell aging has not been reported yet. Supporting the role of *HSP90B1* in aging, it has been documented to be increased in aged rat hippocampi [49], and HSP90 inhibitors were selected in a screen as potential senolytic drugs that intervene in cellular senescence [50]. These results, together with our findings, suggest that *HSP90B1* may serve as a hallmark of aging β-cells, and provide a potential target to modulate β-cell function for a better glucose response. As *HSP90B1* is upregulated with other components of the UPR pathway that have been identified in our dataset, it will be interesting to test other regimens to conquer aging-related deterioration of glucose tolerance by targeting additional UPR components. In summary, the single-cell transcriptional atlas of aged pancreatic islets mapped here indicates that loss of proteostasis is a hallmark of aged β-cells and that selectively targeting specific UPR pathways may restore insulin secretion and glucose homeostasis, perhaps thereby delaying the onset of diabetes.

## MATERIALS AND METHODS

Information on materials used to conduct the research, and all methods used in the analysis are available in the supplementary information.

## Supplementary Material

nwaa127_Supplement_Files
