# Supplementary material for: A single-cell transcriptomic atlas of primate pancreatic islet aging
Source: Natl Sci Rev. 2020 Jun 10;8(2):nwaa127. doi: 10.1093/nsr/nwaa127 (PMC8288398; doi:10.1093/nsr/nwaa127)
Supplement: nwaa127_Supplement_Files [file nwaa127_supplement_files.zip › Supplemental_Materials_and_Methods.pdf]

Supplementary Information

Supplemental Materials and Methods

Animals
Ethics Review Committee approval for research use of cynomolgus tissue was obtained from the Institute of Zoology of the Chinese Academy of Sciences in advance (1). Originated from Southeast Asia, eight young (4-6 years old) and eight old (18-21 years old) *Macaca fascicularis* were raised at Xieerxin Biology Resource under careful supervision, an accredited primate research center in Beijing. The monkeys were kept at ~25°C with a 12-h light/dark cycle and were fed with a semi-purified, nutritionally fortified diet supplemented with fruit. All animals were excluded from a history of clinical disorder and/or prior experimental use. Somatometric data collected for each monkey showed an increase in body weight and body mass index (BMI) in the old group in both males and females, consistent with normative age- and gender-related changes in monkey body composition (2). Blood tests were performed to ensure that there was no indication of hyper- or hypo-glycemia, or pancreatic diseases. Detailed information about the animals analyzed in this study is included in Supplementary Fig. S1A.

Pancreatic Islet Isolation and Cell Dissociation
Tissues were collected at the same time at each day over 16 sequential days. The animals were anesthetized, followed by perfusion with physiological saline, and the whole pancreas was isolated. 1/3 of the pancreas near the tail was utilized to isolate pancreatic islets. The tail part was ligated with surgical sutures, perfused with 20 ml 0.5 mg/ml Collagenase V (STEMCELL, 07431) in HBSS (Gibco, 14025), and incubated at 37°C for 35 min. Digestion was neutralized with 25 ml 10% FBS (Gibco, 10270-106) in HBSS. Islets were isolated by centrifugation at 4℃ for 1 min at 1,000 rpm, rewashed with 10 ml 10% FBS in HBSS once, and collected in 10 ml HBSS. The collected solution was then transferred into a 3.5-cm dish for the enrichment of pancreatic islets by hand-picking under microscopy. Then cells were dissociated by incubation with Accumax (Millipore, SCR006) at 37℃ for 5-10 min, stopped using 10% FBS in HBSS and collected in 2 ml HBSS. The cell suspension was incubated with propidium iodide (PI) (Invitrogen, P3566), followed by removal of debris using fluorescence-activated cell sorting (FACS) (BD FACSAria™ II). Cells were then subjected to single-cell library construction.

Single-Cell RNA-Seq (scRNA-seq) Library Construction and Sequencing
A modified STRT-seq protocol with modifications was utilized to generate scRNA-seq libraries as previously described (1, 3, 4). Briefly, 384 single pancreatic islet cells were picked into lysis buffer by mouth pipetting for each monkey. mRNA in lysates was then reverse transcribed with SuperScript II reverse transcriptase (Invitrogen, 18064-071), and an 8-nt cell-specific barcode was added to cDNA from each cell. Absolute counting of molecules was performed with 8-nt unique molecular identifiers (UMIs). The synthesized cDNA from different cells was amplified with 15 cycles and pooled together, followed by fragmentation with Covaris S2 and enrichment of specific fragments using Dynabeads MyOne Streptavidin C1 beads (Thermo, 65001). The enriched fragments were constructed into libraries using KAPA Hyper Prep Kit (KAPA, KK8504), and sequenced on an Illumina HiSeq X Ten platform by Novogene.

Immunostaining

A third of the tissue near the head of the pancreas was fixed in 4% paraformaldehyde (PFA) (Dingguo, AR-0211) at 4℃ overnight, washed extensively with phosphate-buffered saline (PBS), soaked in 30% sucrose, embedded in paraffin wax and cut into 5-μm-thick sections. As PFA-fixed tissues of one young female and one old male monkey were not processed properly due to limited experimental experience at the beginning, PFA-fixed tissues of 14 monkeys were utilized for evaluation. The paraffin tissues sections were deparaffinized and rehydrated followed by antigen retrieval. Sections were blocked with 10% donkey serum in PBS for 1 h at room temperature and incubated with primary antibodies at 4℃ overnight. For immunochemical analysis of insulin, ERO1LB and TMEM132B in Fig. 1E, the sections were stained using an IHC kit (ZSBIO, PV-9001, PV-9002, ZLI-9018) following the manual. The primary antibodies used were mouse anti-insulin (Sigma, I2008, 1:200), rabbit anti-ERO1B (Proteintech, 11261-2-AP, 1:100), rabbit anti-TMEM132B (Novus Biologicals, NBP2-38259, 1:200). For immunofluorescence staining of other marker proteins, sections were incubated with fluorescence-labeled secondary antibodies (diluted with 1% donkey serum in PBS) at room temperature for 1 h. Hoechst 33342 (Invitrogen) was used to stain nuclear DNA. The primary antibodies used were mouse anti-insulin (Sigma, I2008, 1:200), guinea pig anti-insulin (DAKO, IR00261, 1:1), mouse anti-glucagon (Abcam, ab10988, 1:200), rabbit anti-somatostatin (Millipore, MAB354, 1:200), rabbit anti-ERO1B (Proteintech, 11261-2-AP, 1:100). A confocal laser-scanning microscope (Leica TCS SP5 II) was used to obtain images.

The remainder of the pancreatic tissue was cut into small pieces, embedded in Tissue-Tek® O.C.T.™ Compound (Sakura Finetek, 4583) and stored in liquid nitrogen. The frozen OCT tissues were cut into 10-μm-thick sections. Pancreatic sections from 16 monkeys were fixed with 4% PFA in PBS for 30 min, permeabilized with 0.4% Triton X-100 (Sigma, T9284) in PBS for 30 min, and blocked with 10% donkey serum in PBS for 1 h. Then the sections were incubated overnight with primary antibodies at 4°C overnight, and then incubated with fluorophore-labeled secondary antibodies (diluted with 1% donkey serum in PBS) at room temperature for 1 h. As above, Hoechst 33342 (Invitrogen) was used to stain nuclear DNA. The primary antibodies used were mouse anti-insulin (Sigma, I2008, 1:200) and rat anti-HSP90B1 (Thermo, MA3-016, 1:200). Sections were stained and imaged in parallel such that the staining intensity reflected protein expression. Images were captured systematically covering the whole section in confocal mode on a Leica SP5 microscope. The Opera Phenix high-content imaging system (PerkinElmer, Massachusetts, USA) was utilized for quantification of the mean fluorescence intensities of HSP90B1 in β-cells. The intensities of INS-positive cells were measured in 13-132 (average 55.6) different fields in each section, and normalized to those in the young female group.

Aggresome Visualization

For analysis of aggresome intensity, frozen sections were stained with PROTEOSTAT® aggresome detection kit (ENZO, ENZ-51035-K100). Briefly, sections were fixed using 4% PFA in PBS for 15 min, permeabilized with 0.3% Triton X-100 (Sigma, T9284) in PBS for 7 min, incubated with the aggresome dye (1:2000 dilution in PBS) for 3 min, and destained in 1% acetic acid for 20 min. After washing thoroughly with PBS, sections were blocked with 10% donkey serum in PBS for 1 h. The sections were then sequentially incubated with mouse anti-insulin antibody (Sigma, I2008, 1:200), fluorescence-labeled secondary antibody, and Hoechst 33342. Images were obtained by confocal laser-scanning system (Leica TCS SP5 II). Aggresome intensity was quantified by ImageJ software and normalized to those of young female group. For each monkey, a total of 5-25 islets were examined.

Hematoxylin and Eosin (H&E) Staining

For H&E staining, paraffin sections were stained according to standard methods as previously described (1). The number and average area of aged pancreatic islets were quantified using ImageJ software and normalized to those of the young group. Every islet (eight or more cells) was evaluated. The number of islets was normalized by the area of the section. The area of 80-250 islets was measured for each animal. The differences in the proportions of β-cells between ones captured by H&E staining and scRNA-seq data might stem from the low survival rate of β-cells during isolation procedure (5).

TUNEL Staining
For TUNEL staining, paraffin sections were stained using the *in Situ* Cell Death Detection Kit, POD (Roche, 11684817910) following the manufacturer's protocol, then counterstained with hematoxylin solution for visualization of the nucleus. Average percentages of TUNEL-positive cells over 18-26 islets for each animal were quantified using ImageJ software and normalized to those of the young group.

Blood and Plasma Assay
Blood glucose of 16 monkeys was measured using the OneTouch® UltraVue™ Blood Glucose Monitoring System. Plasma C-peptide, insulin and glucagon were determined by Iodine[125I] C-peptide Radioimmunoassay Kit (BNIBT, S10940099), Iodine[125I] Insulin Radioimmunoassay Kit (BNIBT, S10930046) and Iodine[125I] Glucagon Radioimmunoassay Kit (BNIBT, S10950157) according to the manufacturer's instruction. For the plasma assay, four young male, four old female, four old male, and two young female monkeys were measured, as we failed to extract enough blood by phlebotomizing from the other two young female monkeys.

Cell Culture
Mouse MIN6 islet β-cells were kindly provided by Prof. Tao Xu (Institute of Biophysics, Chinese Academy of Sciences, China). Mouse MIN6 islet β-cells were cultured in Dulbecco's modified Eagle's medium (DMEM, 25 mM glucose) (HyClone, SH30243.02), supplemented with 10% FBS (Gibco, 10270106), 1 × 2-Mercaptoethanol (Gibco, 21985-023), 1% penicillin/streptomycin (Gibco, 15140122) and 0.01% plasmocin (InvivoGen, ant-mpt). No contamination of mycoplasma was observed during cell culture. Mycoplasma contamination was detected by PCR-based method. Briefly, the culture medium was sampled after 24 h of incubation and examined by PCR along with a positive control. After agarose gel electrophoresis, mycoplasma-positive samples will yield a fragment of approximately 250 bp.

Glucose-Stimulated Insulin Secretion (GSIS) Assay
MIN6 islet β-cells were cultured in 5% CO_2 at 37℃ in 12-well plates. After two days of culture, the culture medium was replaced with 0 mM glucose Krebs-Ringer Bicarbonate (KRB) buffer (0.2% BSA in KRB buffer), and washed twice. The supernatant was collected after incubation with 0 mM glucose KRB buffer for 30 min and repeated three times. Then cells were washed once and incubated with 16.7 mM glucose KRB buffer for 30 min. The supernatant was collected for insulin analysis by Insulin ELISA kit (Abcam, ab100578) according to the manufacture's recommendation.

Lentivirus Packaging
Lentiviral overexpression vectors were constructed by cloning human *HSP90B1* cDNA into the pLE4 vector (a gift from Dr. Tomoaki Hishida). For lentiviral generation, HEK 293T cells were co-transfected with a lentivirus overexpression vector, packaging plasmids psPAX2 (Addgene, 12260) and pMD2.G (Addgene, 12259) using Lipofectamine 3000 (Invitrogen). Lentiviral

particles were collected by ultracentrifugation at 19,400 g for 2.5 h. Primers used for vector construction are provided in Supplementary Table 5.

RNA and Protein Analysis
For quantitative PCR analysis, the total RNA was extracted by the TRIzol (Thermo) and reverse transcribed to cDNA using the GoScript Reverse Transcription System (Promega). Quantitative PCR was performed using iTaq Universal SYBR Green Super mix (Bio-Rad) on a CFX384 Real-Time PCR system (Bio-Rad). Data were normalized to mouse Actin gene. Primers used for RT-qPCR were provided in Supplementary Table 5. For western blots, cells were harvested and lysed in 2 × SDS, and quantified using a BCA Kit (Dingguo, BCA-02). Western blot was conducted as previously described (6).

Processing Single-Cell RNA-Seq Data
Single-cell RNA-seq raw sequencing data were first trimmed to remove the TSO and poly A tail sequence, and reads with low-quality bases, as well as those contaminated with adapters, were discarded using a custom script. Clean reads were then aligned to the Ensembl *Macaca fascicularis* reference genome (version: Macaca_fascicularis_5.0) using *Tophat* (version: 2.0.12) (7). UMI counts of uniquely mapped reads were counted using the *htseq-count* tool from *HTSeq* (8), and transcripts with the same UMI were only counted once. The gene expression level was quantified as transcripts per million (TPM). Since the scRNA-seq library complexity was estimated to be ~100,000 transcripts, gene expression levels were normalized into Log_2(TPM/10 + 1).

To retain single cells with high quality, three measures were applied: the rate of reads mapping into the reference genome was more than 30%, the number of genes detected in the annotation genome was more than 800, and the number of UMIs more than 10,000 (Supplementary Fig. S2A). After this critical cell filtering strategy, 5,575 of 6,141 single cells were retained for the downstream analysis.

Identification of Cell Types and Cell-Type-Specific Marker Genes
Given the potential individual heterogeneity and batch effects, raw data of gene expression levels were normalized using the algorithm of mutual nearest neighbors (MNN) (9). The MNN correction was applied using function *mnnCorrect* in R package *scran* (version: 1.2.2) with parameters '*cos.norm.in=TRUE, cos.norm.out=TRUE, var.adj=TRUE, k=20, sigma=0.1*' (10). Based on the corrected gene expression data, the distance matrix was obtained using function *dist* with default parameters. Then, the distance matrix was utilized to perform the *t*-distributed stochastic neighbor embedding (*t*-SNE) analysis (Fig. 1B; Supplementary Fig. S2B) using function *Rtsne* in R package *Rtsne* (version: 0.13) with parameters '*is_distance=TRUE, perplexity=30*', and single cells were classified with the unsupervised clustering method using the command *hclust(as.dist(1-abs(cor(tmp.corrected.df, method='pearson'))), method='ward.D2').*

To verify the robustness of the clustering strategy, the above normalization was performed with cells collected from only young or aged individuals. The *t*-SNE analysis and unsupervised clustering analysis were performed as described above. The circos plot was generated using the function *chordDiagram* in R package *circlize* (version: 0.4.6).

Based on the raw gene expression TPM data, two-tailed Student's *t*-test was used to identify cell-type-specific marker genes (differentially expressed genes among different cell types) using the function *FindAllMarkers* in R package *Seurat* (version: 1.4.0.14) (11). Marker genes

were selected only if three standards were matched: Log_2-transferred average difference was greater than 1, *P*-value was less than 0.05, and the percentage of gene-expressing cells in the corresponding cluster was greater than 25%. A total of 205, 231, 147 and 151 cell-type-specific marker genes were identified in α-cells, β-cells, δ-cells and PP-cells, respectively. To show the marker gene in the heatmap, the average expression level of each marker gene in a given cell type and a given individual was calculated (Fig. 1D). The expression levels of marker genes were shown in *t*-SNE plots or bar plots (Fig. 1B, C and G; Supplementary Fig. S2D). GO analysis of cell-type-specific marker genes was performed with *Metascape* with default parameters (Supplementary Fig. S2C) (12).

To detect the enriched pathway in each cell type, KEGG (Kyoto Encyclopedia of Genes and Genomes) pathway enrichment analysis between a given cell type and the other cell types was performed by gene set enrichment analysis (GSEA). Briefly, GSEA was performed using the Java software *GSEA* (version: 3.0, desktop application) based on KEGG gene sets ('*c2: curated gene sets*') (13). The scoring scheme for hits (gene set numbers) and misses (non-members) was set to the '*weighted*' enrichment statistic method, and the metric for ranking genes was selected with the '*Signal2Noise*' method. To ensure reproducibility, the random seed was set as '*19961109*' in the permutations process. All other parameters to perform GSEA were by default. The pathway enrichment analysis result was shown with the dot plot (Fig. 1F).

Transcriptional Noise Analysis

The analysis method and definition of transcription noise were similar to those used in a previous study (14). Transcriptional noise was defined as biological variation over technology variation using Pearson correlation to assess.

For each gene in a given cell type, the standard deviation, the average value and the coefficient of variation (CV) of the gene expression level were calculated firstly, and then the top-ranked (ranked by CV descending) 1,000 genes with average expression levels more than 1 were selected as highly variable genes (HVGs). Only HVGs were utilized to calculate biological variation in the downstream analysis.

Firstly, the biological variation $b_{c,i,k}$ for each cell was defined as

$$b_{c,i,k} = 1 - \mathrm{cor}(b\_exp_{c,i,k} - b\_mean_{c,i}),$$

where $b\_exp_{c,i,k}$ indicated the endogenous expression vector in cell k of cell type c and monkey individual i, and $b\_mean_{c,i}$ indicated the endogenous average expression vector in cell type c and monkey individual i. Secondly, the technology variation $t_{c,i,k}$ for each cell was defined as

$$t_{c,i,k} = 1 - \mathrm{cor}(t\_exp_{c,i,k} - t\_mean),$$

where $t\_exp_{c,i,k}$ indicated the spike-in expression vector in cell k of cell type c and monkey individual i, and t_mean indicated the spike-in average expression vector in all filtered cells (5,575 filtered cells in total). Thirdly, transcriptional noise was defined as the ratio of biology variation and technology variation for the given cell type.

Identification of Young and Old Cells

To identify young and old cells based on transcriptomic data, principal component analysis (PCA) was performed to investigate the gene set associated with the aging process. HVGs (647 HVGs for α-cells and 2,213 HVGs for β-cells) were firstly selected using function *MeanVarPlot* in R package *Seurat*. PCA was performed with these selected HVGs using function *PCA* in *Seurat*. Several top-ranked principal components (PCs) obtained from PCA were associated with individual heterogeneity or other factors (PC1~PC8 for α-cells and PC1~PC3 for β-cells), and there was one PC associated with the aging process (PC9 for α-cells and PC4 for β-cells)

(Supplementary Fig. S4A).

Young and old cells were defined by combing information from the principal component and the age group information of each single cell. For example, for α-cells, young cells (650 cells collected from male individuals, 793 from female individuals, 1,443 cells in total) were defined as cells collected from young monkey individuals and distributed in the positive axis of PC9, and old cells (male: 910, female: 487, in total: 1,397) were defined as cells collected from aged monkey individuals and distributed in the negative axis of PC9; for β-cells, young cells (male: 197, female: 172, in total: 369) were defined as cells collected from young monkey individuals and distributed in the negative axis of PC4, and old cells (male: 227, female: 219, in total: 446) were defined as cells collected from aged monkey individuals and distributed in the positive axis of PC4. These identified young and old cells were used in the downstream analysis.

When we separately identified aging-associated differentially expressed genes (DEGs) in male or female monkey individuals, we directly used the above identified young and old cells.

Identification of Aging-Associated Differentially Expressed Genes

Aging-associated DEGs were identified in the young and old cells (we called 'PCA strategy' in this study, Supplementary Fig. S4D). For α-cells, there were 1,443 young cells and 1,397 old cells; for β-cells, there were 369 young cells and 446 old cells. The two-tailed Student's *t*-test classifier was used to identify aging-associated DEGs using the function *FindMarkers* in R package *Seurat*. DEGs were selected only if three standards were matched: Log_2-transferred average difference was greater than 0.5, *P*-value was less than 0.05, and the percentage of gene-expressing cells in the corresponding age group was greater than 25%. GO analysis was performed with *Metascape* with default parameters.

To validate the reliability of DEGs obtained from young and old cells, the other DEG gene set was obtained with the additional analysis (we called 'direct strategy' in this study, Supplementary Fig. S4B-D), in which we obtained aging-associated DEGs by comparing all cells collected from young and old monkeys. For example, for α-cells, we used 2,168 cells collected from young monkeys and 2,014 cells from old monkeys to calculate aging-associated DEGs; for β-cells, we used 524 cells from young monkeys and 645 cells from old monkeys to calculate aging-associated DEGs. We identified aging-associated DEGs with the same method described as above.

Cell-Cell Interaction Analyses

The cell-cell interaction analysis was performed based on single-cell RNA-seq data (gene expression levels were quantified with TPM) using software *CellPhoneDB* (version: 2.0.0) (15). For a special cell type, only the ligand and receptor interacting pairs satisfying the following three conditions were considered in the downstream analysis: (1) at least 10% cells expressed that pair (set the parameter as '*--threshold 0.1*'), (2) the average expression level was greater than 5, and (3) compared between different cell types, the enrichment of the pair was statistically significant ($P < 0.05$). A ligand-receptor pair only detected in cells collected from young individuals was considered as a young-specific interaction pair; a ligand-receptor pair only detected in cells from old individuals was as an old-specific interaction pair; the rest were common interaction pairs.

Statistical Analyses

All bar charts were presented as the mean ± SEM. All statistical analyses were performed by two-tailed Student's *t*-test with the *P* value indicated in each figure. $P < 0.05$ was considered

statistically significant.

Data Availability

All RNA-seq sequencing data and processed data have been deposited in the NCBI Gene Expression Omnibus (GEO) under the accession number GSE120180.

SUPPLEMENTAL REFERENCES

1. Wang, S, Zheng, Y, Li, J*, et al.* Single-Cell Transcriptomic Atlas of Primate Ovarian Aging. *Cell*. 2020; **180**(3): 585-600.e19.
2. Hudson, JC, Baum, ST, Frye, DMD*, et al.* Age and sex differences in body size and composition during Rhesus monkey adulthood. *Aging Clinical and Experimental Research*. 1996; **8**(3): 197-204.
3. Dong, J, Hu, Y, Fan, X*, et al.* Single-cell RNA-seq analysis unveils a prevalent epithelial/mesenchymal hybrid state during mouse organogenesis. *Genome Biol*. 2018; **19**(1): 31.
4. Cui, Y, Zheng, Y, Liu, X*, et al.* Single-Cell Transcriptome Analysis Maps the Developmental Track of the Human Heart. *Cell Reports*. 2019; **26**: 1934-50.e5.
5. Segerstolpe, Å, Palasantza, A, Eliasson, P*, et al.* Single-Cell Transcriptome Profiling of Human Pancreatic Islets in Health and Type 2 Diabetes. *Cell metabolism*. 2016; **24**(4): 593-607.
6. Zhang, W, Li, J, Suzuki, K*, et al.* A Werner syndrome stem cell model unveils heterochromatin alterations as a driver of human aging. *Science*. 2015; **348**(6239): 1160-3.
7. Trapnell, C, Pachter, L, Salzberg, SL. TopHat: discovering splice junctions with RNA-Seq. *Bioinformatics*. 2009; **25**(9): 1105-11.
8. Anders, S, Pyl, PT, Huber, W. HTSeq—a Python framework to work with high-throughput sequencing data. *Bioinformatics*. 2015; **31**(2): 166-9.
9. Haghverdi, L, Lun, AT, Morgan, MD*, et al.* Batch effects in single-cell RNA-sequencing data are corrected by matching mutual nearest neighbors. *Nature biotechnology*. 2018; **36**(5): 421-7.
10. Lun, AT, McCarthy, DJ, Marioni, JC. A step-by-step workflow for low-level analysis of single-cell RNA-seq data with Bioconductor. *F1000Research*. 2016; **5**.
11. Satija, R, Farrell, JA, Gennert, D*, et al.* Spatial reconstruction of single-cell gene expression data. *Nature biotechnology*. 2015; **33**(5): 495-502.
12. Zhou, Y, Zhou, B, Pache, L*, et al.* Metascape provides a biologist-oriented resource for the analysis of systems-level datasets. *Nature communications*. 2019; **10**(1): 1-10.
13. Subramanian, A, Tamayo, P, Mootha, VK*, et al.* Gene set enrichment analysis: a knowledge-based approach for interpreting genome-wide expression profiles. *Proceedings of the National Academy of Sciences*. 2005; **102**(43): 15545-50.
14. Enge, M, Arda, HE, Mignardi, M*, et al.* Single-cell analysis of human pancreas reveals transcriptional signatures of aging and somatic mutation patterns. *Cell*. 2017; **171**(2): 321-30. e14.
15. Vento-Tormo, R, Efremova, M, Botting, RA*, et al.* Single-cell reconstruction of the early maternal–fetal interface in humans. *Nature*. 2018; **563**(7731): 347-53.
