# Supplementary material for: A single-cell transcriptomic atlas of primate pancreatic islet aging
Source: Natl Sci Rev. 2020 Jun 10;8(2):nwaa127. doi: 10.1093/nsr/nwaa127 (PMC8288398; doi:10.1093/nsr/nwaa127)
Supplement: nwaa127_Supplement_Files [file nwaa127_supplement_files.zip › Supplementary_Information.pdf]

Supplementary Information

Supplementary Figures

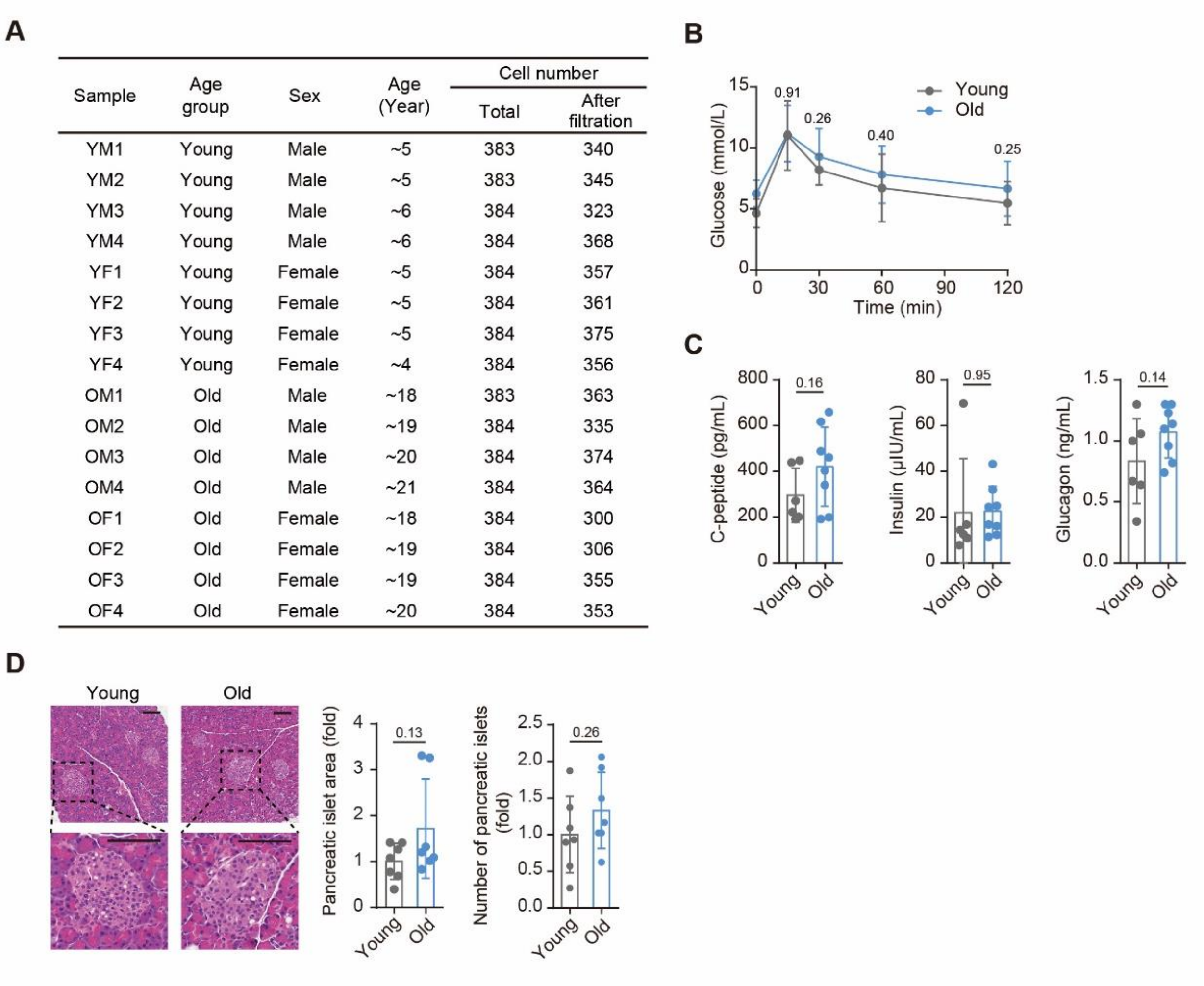

Sample	Age group	Sex	Age (Year)	Cell number: Total	Cell number: After filtration
YM1	Young	Male	~5	383	340
YM2	Young	Male	~5	383	345
YM3	Young	Male	~6	384	323
YM4	Young	Male	~6	384	368
YF1	Young	Female	~5	384	357
YF2	Young	Female	~5	384	361
YF3	Young	Female	~5	384	375
YF4	Young	Female	~4	384	356
OM1	Old	Male	~18	383	363
OM2	Old	Male	~19	384	335
OM3	Old	Male	~20	384	374
OM4	Old	Male	~21	384	364
OF1	Old	Female	~18	384	300
OF2	Old	Female	~19	384	306
OF3	Old	Female	~19	384	355
OF4	Old	Female	~20	384	353

Supplementary Fig. 1. Basic Information of Cynomolgus Monkeys.
(A) A table showing information for cynomolgus monkeys analyzed in this study. The number of sequenced single cells and filtered cells are shown. YM, young male; YF, young female; OM, old male; and OF, old female.
(B) Line plot showing glucose levels at indicated time after meal following 12 h-fasting in young and old groups. *n* = 8 monkeys for each group. *P* values are indicated (two-tailed *t*-test). Data are shown as mean ± SEM.
(C) Bar charts showing the c-peptide level (left), insulin level (middle) and glucagon level (right) of young and old groups. Young, *n* = 6; old, *n* = 8 monkeys. *P* values are indicated (two-tailed *t*-test). Data are shown as mean ± SEM.
(D) H&E-staining of young and old pancreases. Pancreatic islets are circled with dashed lines (left). Bar chart showing the relative pancreatic islet area and relative number of pancreatic islets in young and old monkeys (right). The number and average area of pancreatic islets are normalized to those in the young group. Scale bar, 100 μm. *n* = 7 monkeys for each group. *P* values are indicated (two-tailed *t*-test). Data are shown as mean ± SEM.

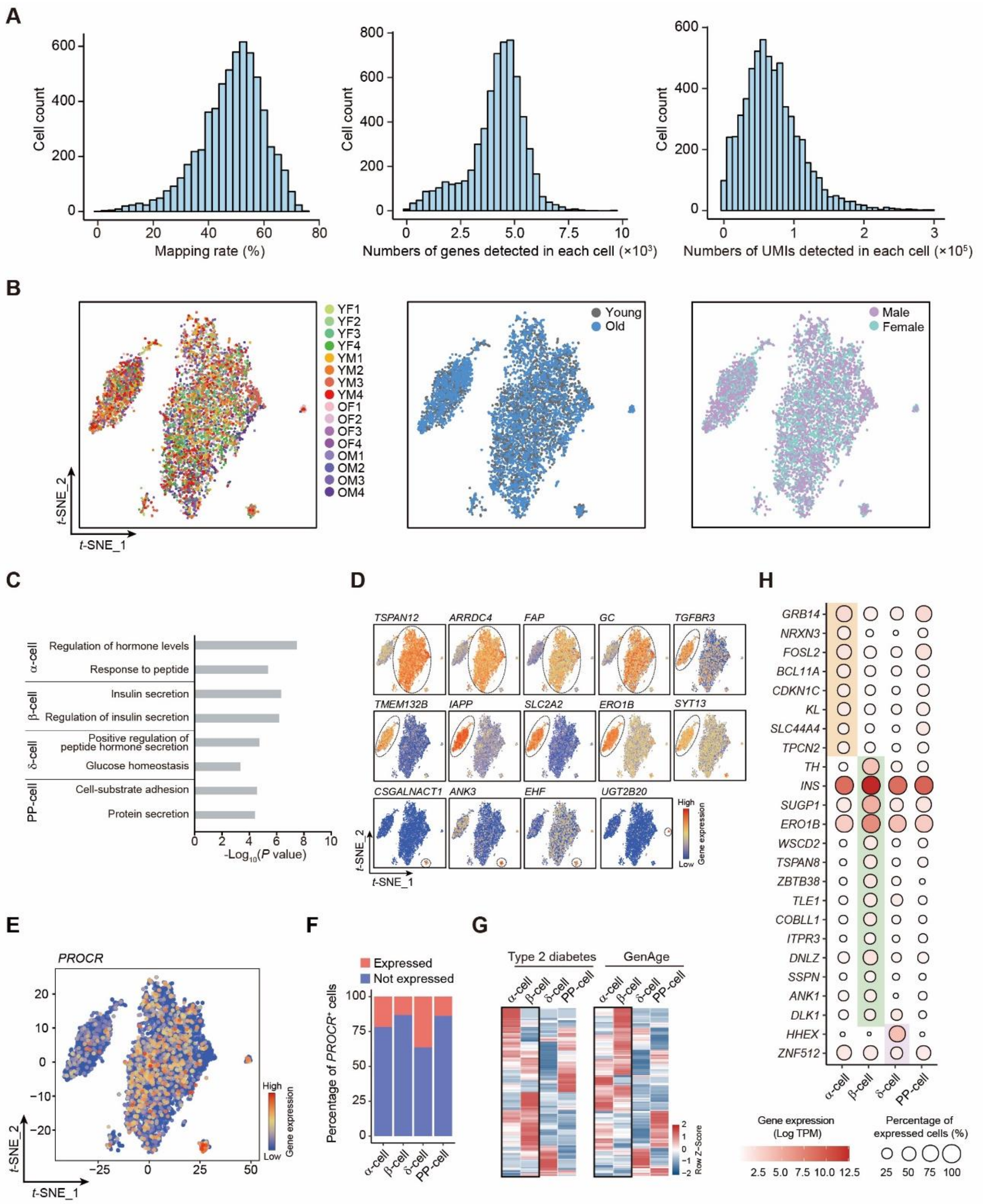

A
Cell count
Mapping rate (%)
Numbers of genes detected in each cell (×10³)
Numbers of UMIs detected in each cell (×10⁵)
B
YF1
YF2
YF3
YF4
YM1
YM2
YM3
YM4
OF1
OF2
OF3
OF4
OM1
OM2
OM3
OM4
Young
Old
Male
Female
t-SNE_1
t-SNE_2
C
α-cell
Regulation of hormone levels
Response to peptide
β-cell
Insulin secretion
Regulation of insulin secretion
δ-cell
Positive regulation of peptide hormone secretion
Glucose homeostasis
PP-cell
Cell-substrate adhesion
Protein secretion
-Log10(P value)
D
TSPAN12
ARRDC4
FAP
GC
TGFBR3
TMEM132B
IAPP
SLC2A2
ERO1B
SYT13
CSGALNACT1
ANK3
EHF
UGT2B20
High
Low
Gene expression
E
PROCR
F
Expressed
Not expressed
Percentage of PROCR+ cells
α-cell
β-cell
δ-cell
PP-cell
G
Type 2 diabetes
GenAge
Row Z-Score
H
GRB14
NRXN3
FOSL2
BCL11A
CDKN1C
KL
SLC44A4
TPCN2
TH
INS
SUGP1
ERO1B
WSCD2
TSPAN8
ZBTB38
TLE1
COBLL1
ITPR3
DNLZ
SSPN
ANK1
DLK1
HHEX
ZNF512
Gene expression (Log TPM)
2.5 5.0 7.5 10.0 12.5
Percentage of expressed cells (%)
25 50 75 100

Supplementary Fig. 2. Quality Control of Single-Cell RNA-Seq Data.

(A) Histograms showing the mapping rate (left), the numbers of genes (middle) and the numbers of UMIs (right) detected in each cell.

(B) *t*-SNE plots showing the distribution of single cells in different monkeys (left), age groups (middle) and sex groups (right).

(C) Bar chart showing representative GO terms of cell-type-specific marker genes in each cell type.

(D) *t*-SNE plots showing expression levels of representative cell-type-specific marker genes. The corresponding cell type is denoted in circles.

(E) *t*-SNE plot showing expression levels of *PROCR*.

(F) Stacked histogram showing the percentages of *PROCR*$^+$ cells and *PROCR*$^-$ cells in each cell type.

(G) Heatmaps showing the row scaled expression levels of genes in T2D (left) (collected from http://www.type2diabetesgenetics.org) and GenAge (right) gene sets (collected from http://genomics.senescence.info/genes) in each cell type.

(H) Dot plot showing expression characteristics of common genes between diabetes associated genes and cell-type-specific marker genes. Color indicates the average gene expression level (Log_2-transferred TPM) and size indicates the percentage of expressed cells. Cell-type-specific marker genes in the corresponding cell type are highlighted.

A

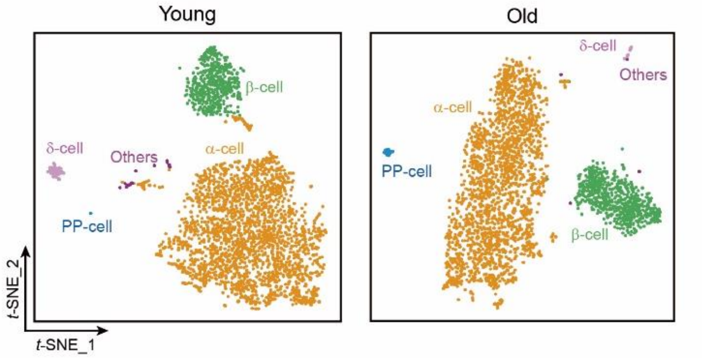

B

Sample	α-cell	β-cell	δ-cell	PP-cell	Other	Sum
YF1	270	65	17	1	4	357
YF2	341	10	5	0	5	361
YF3	331	30	11	0	3	375
YF4	261	85	5	1	4	356
YM1	252	71	6	6	5	340
YM2	217	114	10	4	0	345
YM3	243	43	14	0	23	323
YM4	253	106	2	7	0	368
Sum	2,168	524	70	19	44	2,825
Percentage	76.74%	18.55%	2.48%	0.67%	1.56%	100.00%
OF1	149	133	6	7	5	300
OF2	214	61	9	21	1	306
OF3	326	27	0	1	1	355
OF4	167	184	1	0	1	353
OM1	276	82	3	0	2	363
OM2	261	63	5	5	1	335
OM3	295	73	3	1	2	374
OM4	326	22	10	4	2	364
Sum	2,014	645	37	39	15	2,750
Percentage	73.24%	23.45%	1.35%	1.42%	0.55%	100.00%

C

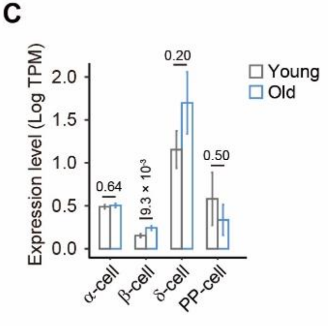

D

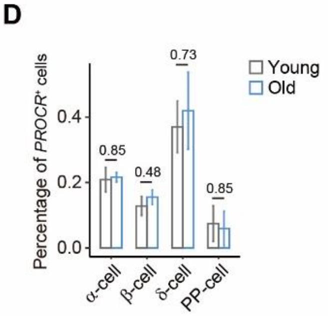

E

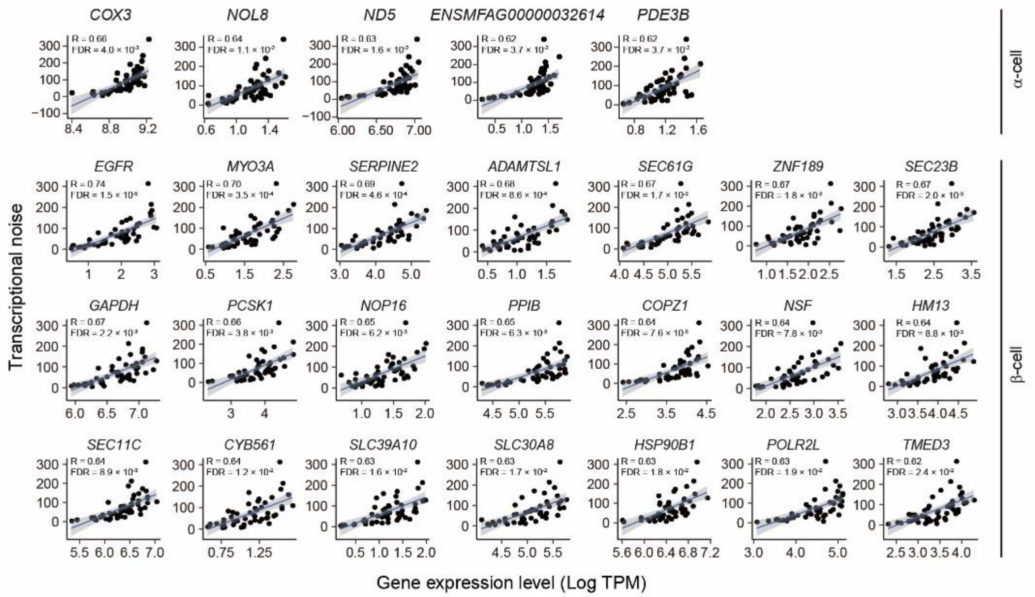

Supplementary Fig. 3. Comparisons of Cell Proportion of Different Types in Young and Old Monkeys.

(A) *t*-SNE plots showing pancreatic islet cell types in young (left) and old (right) monkeys.

(B) A table showing the cell numbers and percentages of each cell type in different monkeys.

(C) Bar chart showing the *PROCR* expression level in each cell type. *P* values are indicated (two-tailed *t*-test). Data are shown as mean ± SEM.

(D) Bar chart showing the percentage of $PROCR^{+}$ cells in each cell type. *P* values are indicated (two-tailed *t*-test). Data are shown as mean ± SEM.

(E) Scatter plots showing the transcriptional noise along with expression levels of representative genes. The shadow indicates the 0.95 confidence interval around smooth. Pearson's correlation coefficients (*R*) and statistical significance (FDR) are indicated.

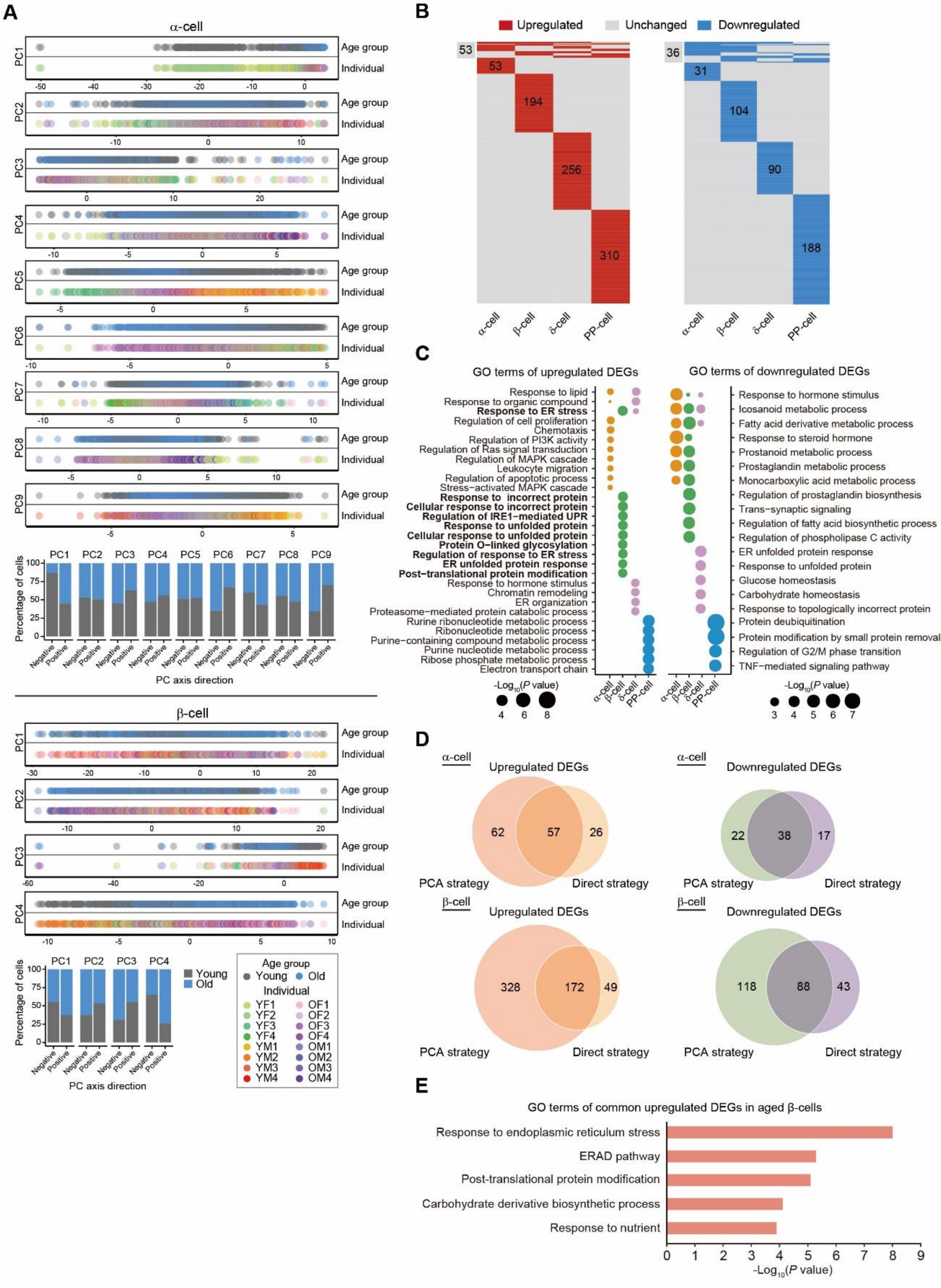
A
α-cell
PC1
PC2
PC3
PC4
PC5
PC6
PC7
PC8
PC9
Age group
Individual
Percentage of cells
Negative
Positive
PC axis direction
β-cell
Young
Old
Age group
Individual
YF1
YF2
YF3
YF4
YM1
YM2
YM3
YM4
OF1
OF2
OF3
OF4
OM1
OM2
OM3
OM4
B
Upregulated
Unchanged
Downregulated
53
53
194
256
310
36
31
104
90
188
α-cell
β-cell
δ-cell
PP-cell
C
GO terms of upregulated DEGs
Response to lipid
Response to organic compound
Response to ER stress
Regulation of cell proliferation
Chemotaxis
Regulation of PI3K activity
Regulation of Ras signal transduction
Regulation of MAPK cascade
Leukocyte migration
Regulation of apoptotic process
Stress−activated MAPK cascade
Response to incorrect protein
Cellular response to incorrect protein
Regulation of IRE1−mediated UPR
Response to unfolded protein
Cellular response to unfolded protein
Protein O−linked glycosylation
Regulation of response to ER stress
ER unfolded protein response
Post−translational protein modification
Response to hormone stimulus
Chromatin remodeling
ER organization
Proteasome−mediated protein catabolic process
Rurine ribonucleotide metabolic process
Ribonucleotide metabolic process
Purine−containing compound metabolic process
Purine nucleotide metabolic process
Ribose phosphate metabolic process
Electron transport chain
GO terms of downregulated DEGs
Response to hormone stimulus
Icosanoid metabolic process
Fatty acid derivative metabolic process
Response to steroid hormone
Prostanoid metabolic process
Prostaglandin metabolic process
Monocarboxylic acid metabolic process
Regulation of prostaglandin biosynthesis
Trans−synaptic signaling
Regulation of fatty acid biosynthetic process
Regulation of phospholipase C activity
ER unfolded protein response
Response to unfolded protein
Glucose homeostasis
Carbohydrate homeostasis
Response to topologically incorrect protein
Protein deubiquitination
Protein modification by small protein removal
Regulation of G2/M phase transition
TNF−mediated signaling pathway
$-Log_{10}(P\text{ value})$
D
α-cell
Upregulated DEGs
62
57
26
Downregulated DEGs
22
38
17
β-cell
Upregulated DEGs
328
172
49
Downregulated DEGs
118
88
43
PCA strategy
Direct strategy
E
GO terms of common upregulated DEGs in aged β-cells
Response to endoplasmic reticulum stress
ERAD pathway
Post-translational protein modification
Carbohydrate derivative biosynthetic process
Response to nutrient
$-Log_{10}(P\text{ value})$

Supplementary Fig. 4. Aging-Associated Transcriptomic Changes in α-cells and β-cells Collected from Young and Old Monkeys.

(A) Dot plots showing the cell distribution along different PCA dimensions in α-cells and β-cells. Dots are colored based on age groups (gray for young monkeys and blue for old monkeys) or individual monkeys (16 colors for 16 monkeys). Stacked histograms showing the percentages of cells collected from young (gray) and old (blue) monkeys in different PCA dimensions (positive and negative axes).

(B) Heatmaps showing the distribution of aging-associated DEGs in each cell type. These DEGs are directly obtained from the comparison between cells from young monkeys and those from old monkeys ('direct strategy'). The number of DEGs in each group is indicated.

(C) Dot plots showing representative GO terms of upregulated (left) or downregulated (right) aging-associated DEGs. These DEGs are directly obtained from the comparison between cells from young monkeys and those from old monkeys ('direct strategy'). Colors indicate different cell types and size indicates $-\text{Log}_{10}(P \text{ value})$.

(D) Venn diagrams showing the overlapping distribution of upregulated and downregulated aging-associated DEGs in aged α-cells and β-cells obtained from two different methods. 'PCA strategy' indicates DEGs obtained from the method based on PCA strategy, and 'direct strategy' indicates DEGs directly obtained from the comparison between cells from young monkeys and those from old monkeys based on sampling information.

(E) Bar chart showing representative GO terms of 172 common upregulated aging-associated DEGs in aged β-cells obtained from two different methods (corresponding to Supplementary Fig. S4D).

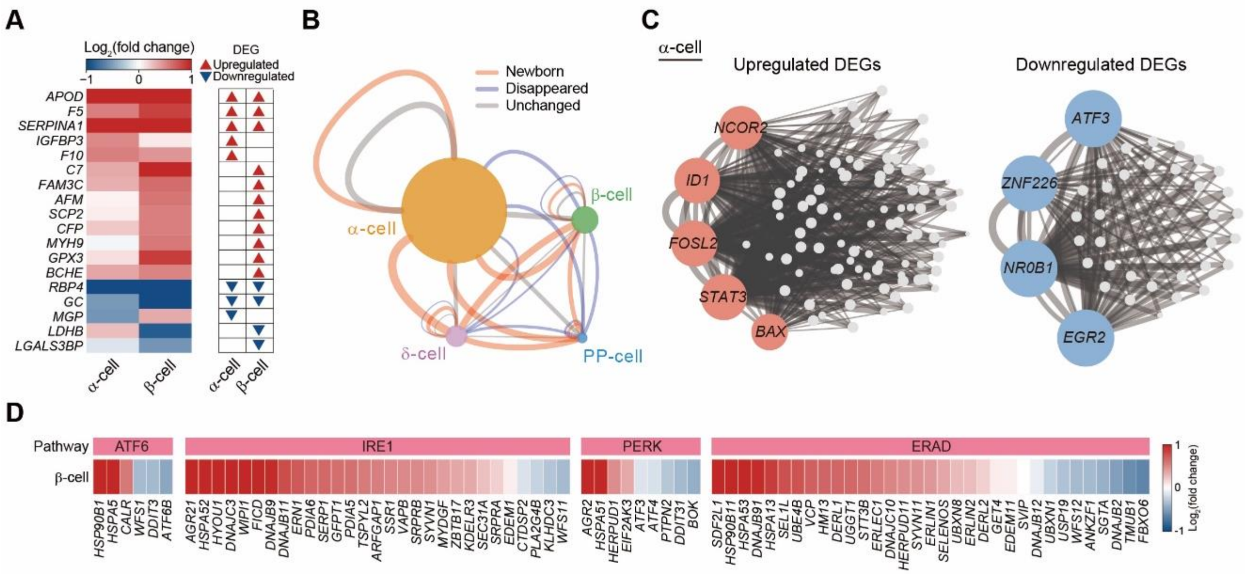

Supplementary Fig. 5. Upregulation of UPR Genes in Aged β-cells.

(A) Heatmap showing the fold change of aging-associated DEGs in plasma (left), and table showing DEG types (right). Red arrows indicate upregulated DEGs and blue arrows indicate downregulated DEGs.

(B) Network showing changes of ligand-receptor interactions in aged monkeys. Each line between any two cell types indicates each ligand-receptor interaction. The node size and line size indicate the number of interactions. Line colors indicate the interaction type during aging process, and red, blue and gray lines correspond to newborn (old-specific), disappeared (young-specific) and unchanged (common) interactions, respectively.

(C) Regulatory network visualizing potential key transcriptional regulators in upregulated and downregulated aging-associated DEGs in aged α-cells. Only connections with high weight are retained and node size indicates the number of connections, and nodes with top-ranked size are highlighted in red (upregulated, left) or blue (downregulated, right).

(D) Heatmaps showing the fold change of genes in four UPR pathways in β-cells. Only the genes differentially regulated in β-cells between young and old individuals are analyzed.

Supplementary Tables

Supplementary Table 1. Quality Control, Cluster Information and Cell-Type-Specific Genes in Single-Cell RNA-Seq Data.
Supplementary Table 2. Aging-Associated Gene Expression Patterns in α-cells and β-cells.
Supplementary Table 3. Aging-Associated Differentially Expressed Genes Identified Based on Sampling Information in Each Cell Type.
Supplementary Table 4. Aging-Associated Differentially Expressed Genes in β-cells of Both Genders.
Supplementary Table 5. Lists of Primers Used in this Study.
